# The Cannabinoid Content of Legal Cannabis in Washington State Varies Systematically Across Testing Facilities and Popular Consumer Products

**DOI:** 10.1038/s41598-018-22755-2

**Published:** 2018-03-14

**Authors:** Nick Jikomes, Michael Zoorob

**Affiliations:** 1 Leafly Holdings, Inc., Division of Data Science, Seattle, WA 98104 USA; 2 Harvard University, Department of Government, Cambridge, MA 02138 USA

## Abstract

The majority of adults in the U.S. now have state-legal access to medical or recreational cannabis products, despite their federal prohibition. Given the wide array of pharmacologically active compounds in these products, it is essential that their biochemical profile is measured and reported to consumers, which requires accurate laboratory testing. However, no universal standards for laboratory testing protocols currently exist, and there is controversy as to whether all reported results are legitimate. To investigate these concerns, we analyzed a publicly available seed-to-sale traceability dataset from Washington state containing measurements of the cannabinoid content of legal cannabis products from state-certified laboratories. Consistent with previous work, we found that commercial *Cannabis* strains fall into three broad chemotypes defined by the THC:CBD ratio. Moreover, we documented systematic differences in the cannabinoid content reported by different laboratories, relative stability in cannabinoid levels of commercial flower and concentrates over time, and differences between popular commercial strains. Importantly, interlab differences in cannabinoid reporting persisted even after controlling for plausible confounds. Our results underscore the need for standardized laboratory methodologies in the legal cannabis industry and provide a framework for quantitatively assessing laboratory quality.

## Introduction

For millennia, *Cannabis* has been cultivated for medicinal, recreational, and industrial purposes^1^. Despite mounting evidence for the legitimate medical utility of cannabis products and their principal psychoactive constituents^2,3^, they remain classified as Schedule I controlled substances by the U.S. federal government. Nonetheless, public opinion on legal cannabis has changed dramatically in recent years^4^ and a majority of U.S. states now allow legal access to medical cannabis for approved patients, with several states also allowing recreational adult-use^5,6^. This dynamic legal landscape has given rise to a rapidly growing legal cannabis industry that offers a wide variety of products to consumers.

Because the core product of this burgeoning industry contains multiple compounds with psychoactive and medicinal properties^7^, it is imperative that the major biochemical constituents of cannabis are accurately quantified, and the results made accessible to consumers. Because recreational cannabis products may differ substantially from cannabis grown for federally-sanctioned research^8^ or found on the black market^9^, there is a particular need to study the commercial cannabis being consumed today by millions of adults in states allowing legal adult-use consumption.

The adoption of universal industry testing standards will be crucial for comparing data across the many existing testing laboratories. However, standardized procedures have yet to be adopted, and controversy exists about whether all laboratories are accurately measuring and reporting cannabinoid content^10^. Most of these labs were not established quality control labs with a track record of testing food or pharmaceutical products, but rather started specifically to focus on cannabis products. At present, there is limited published data^8^ on the content of commercial cannabis products in the U.S., including quantification of potential differences in the measurements reported across these testing laboratories. Reliable testing data will also shed light on questions important to consumers and regulators, such as whether cannabinoid levels are changing over time or differ systematically between commercial products.

To investigate these concerns, we analyzed a large dataset from Washington state’s seed-to-sale traceability system. This dataset comprises hundreds of thousands of measurements of the principal cannabinoids in commercial cannabis, including tetrahydrocannabinol (THC) and cannabidiol (CBD). These measurements are available for commercial products tested across all state-licensed laboratories since 2014, which allowed us to assess the cannabinoid composition of commercial products between laboratories, over time, and across strains.

## Results

### The Basic Chemotype Landscape of Commercial Cannabis

**Figure 1:**
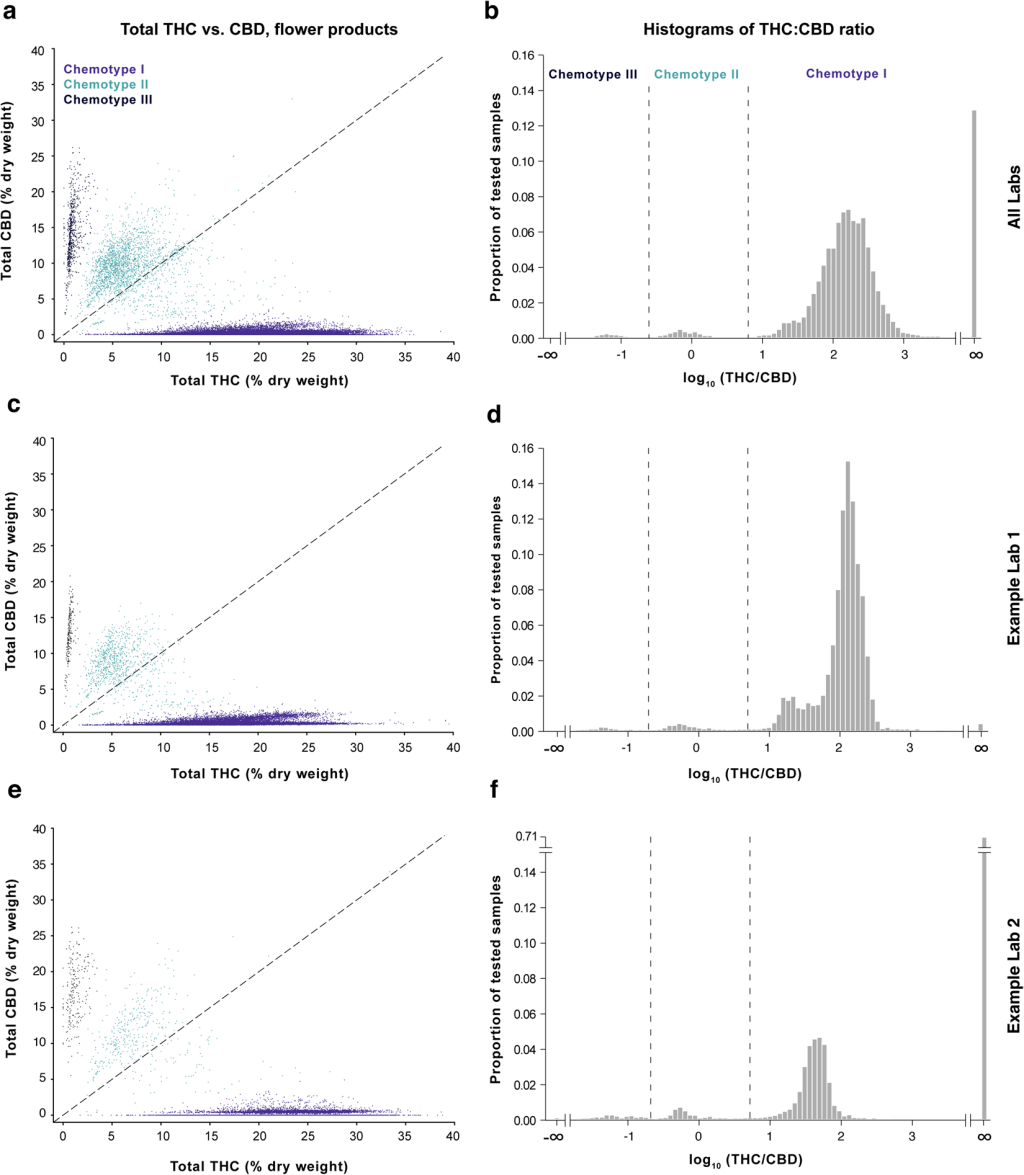
The THC:CBD ratio defines three broad chemotypes of commercial cannabis flower measured by testing labs in Washington. Left column: Scatterplots of total THC vs. total CBD levels for cannabis flower. Right column: Histograms showing the THC:CBD ratio on a log scale and indicating the proportion of flower samples for each chemotype. Data are displayed for measurements batched across all Labs A-F (panels a-b; n = 175,136), for the lab reporting the lowest mean total THC levels (Lab A; panels c-d; n = 62,719), and the lab reporting the highest mean total THC levels (Lab F; panels e-f; n=26,664). Histograms for each of the six labs contributing to batched data in panels a-b are shown in Figure S1. Panels a and c were subsampled to n=50,000 for visualization purposes.

*Cannabis* likely evolved in Central Asia, and landraces native to regions including Afghanistan, Pakistan, India and China^11^ have been found to fall into three general chemotypes based on genetically-constrained THC:CBD ratios^12,13^. Consistent with previous work in landraces and commercial Dutch *Cannabis*^13,14^, we found that commercial *Cannabis* grown in Washington also conforms to this pattern (Fig. 1a–c). Unlike landraces, which are more likely fall into chemotype III (CBD-dominant) category and generally display lower overall levels of total THC^13^, most commercial *Cannabis* falls into the chemotype I category, characterized by relatively high total THC and low total CBD levels (Fig. 1d–f; see Methods for definition of total THC and CBD levels). While studying the chemotype landscape of these commercial samples, we observed striking differences in THC:CBD distributions across laboratories for both flower (Fig. 1d–f, Figure S1) and concentrates (Figure S2). This prompted us to examine interlab differences in more detail. In particular, we wished to assess whether this variation stemmed from intrinsic (e.g. methodological) differences between laboratories or from heterogeneity in the products submitted to those labs.

### THC and CBD Measurements Vary Widely Across Testing Laboratories

**Figure 2:**
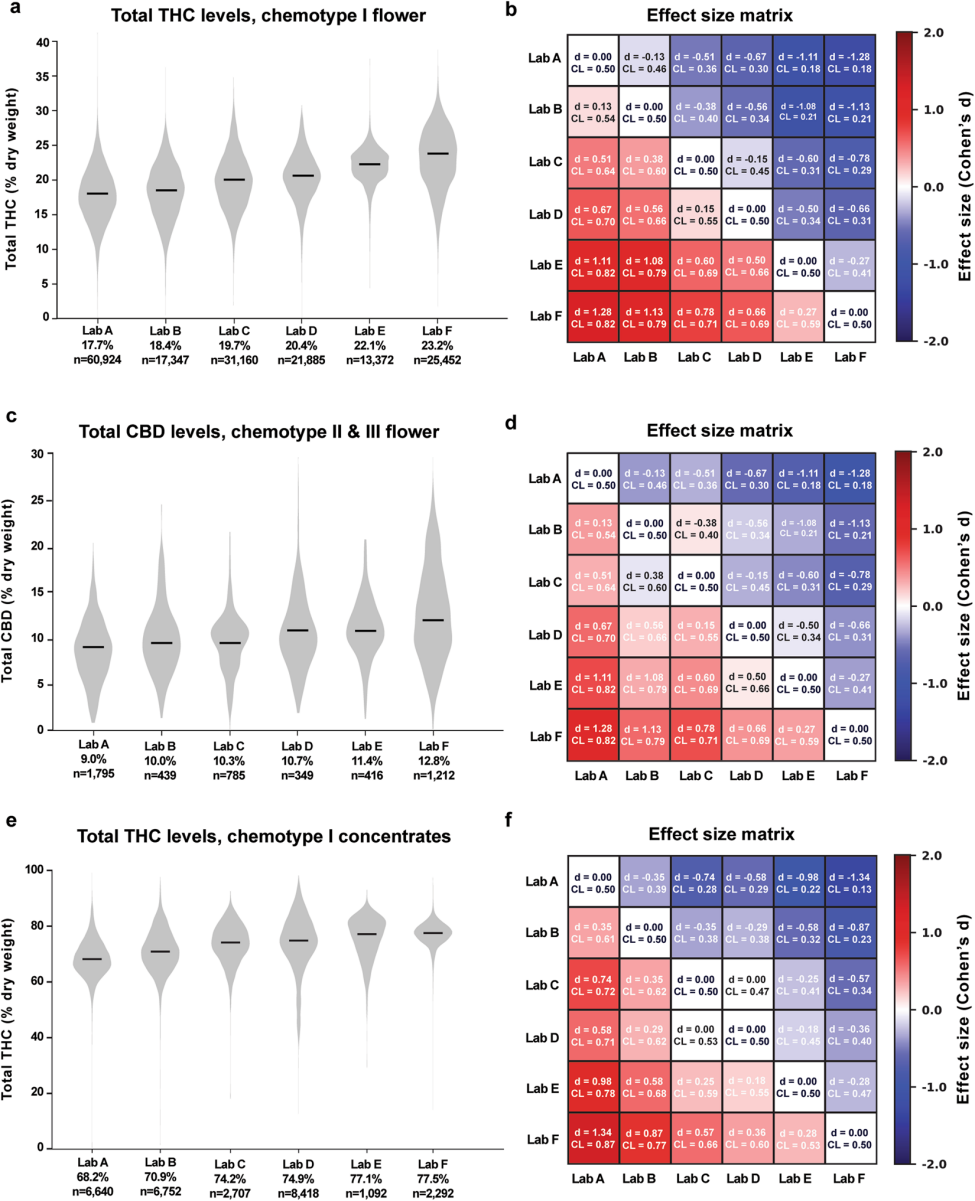
Total THC and CBD Measurements Differ Between Labs Across Chemotypes and Product Categories. Left column: Violin plots showing the distribution of total THC or CBD levels across labs A-F. Black lines denote median values, which are printed below the x-axis for each lab. Right column: Effect size matrices displaying the effect size of pairwise differences in distributions between labs. Matrices are color-coded according to one measure of effect size (Cohen’s d), and a second measure (Common Language) is printed for each comparison.

To compare cannabinoid measurements across labs, we looked at distributions of total THC and CBD levels for the six largest laboratories by data volume for different chemotypes and product categories. These labs, referred to henceforth as labs A-F, are Confidence Analytics (Lab A), Analytical 360 (Lab B), Green Grower Labs (Lab C), Integrity Labs (Lab D), Testing Technologies (Lab E), and Peak Analytics (Lab F). We observed differences in reported values of both THC and CBD (Fig. 2). For example, the median total THC content for chemotype I flower products ranged from 17.7% to 23.2% between the labs reporting the lowest and highest THC levels, respectively (Fig. 2a; labs A-F ordered from lowest to highest median reported THC levels). Pairwise differences in mean THC content between labs were statistically significant (p < 0.001 for each pairwise comparison in Fig. 2a, two-sided t-test). To quantify the magnitude of differences between labs, we calculated the effect sizes of pairwise differences using two metrics: Cohen’s d, the standardized difference between two means^15^, and a “Common Language” (CL) effect size, the probability that a random value from one sample will be greater than a random value from the other^16^ (Fig. 2b; see Analytical Methods).

Calculating effect sizes allows a more intuitive assessment of the magnitude of interlab differences, especially when very large sample sizes allow even trivial differences between means to reach statistical significance. For example, mean THC levels of chemotype I flower for Lab B and Lab A were 18.4% and 17.7%, respectively (Fig. 2a and b). While this difference was highly significant due to the large sample sizes, the effect size was small (d = 0.13; see Methods). The common language effect size (CL) for this comparison was 0.54, indicating a 54% chance that a random THC measurement from Lab B will be larger than a random measurement from Lab A. In contrast, when comparing Lab F to Lab A, which reported the highest mean THC levels, the effect size was considerably larger (d = 1.28, CL = 0.82).

We observed a similar pattern when comparing CBD measurements across labs for chemotype II and III flower samples (Fig. 2c and d) and THC levels for concentrates (Fig. 2e and f). The labs reporting the highest levels of THC for chemotype I flower products also reported the highest levels of CBD for other flower chemotypes and THC levels for concentrates (Fig. 2), indicating a systematic tendency for certain labs to report higher levels of cannabinoids across chemotypes and product categories. This may be explained by differences in laboratory protocols. While most labs report using High Performance Liquid Chromatography (HPLC) to detect cannabinoids, the details of each protocol likely differ. Alternatively, interlab differences may be driven by labs receiving distinct sets of cannabis products for testing.

### Interlab Differences Persist After Controlling for Plausible Confounds

**Figure 3:**
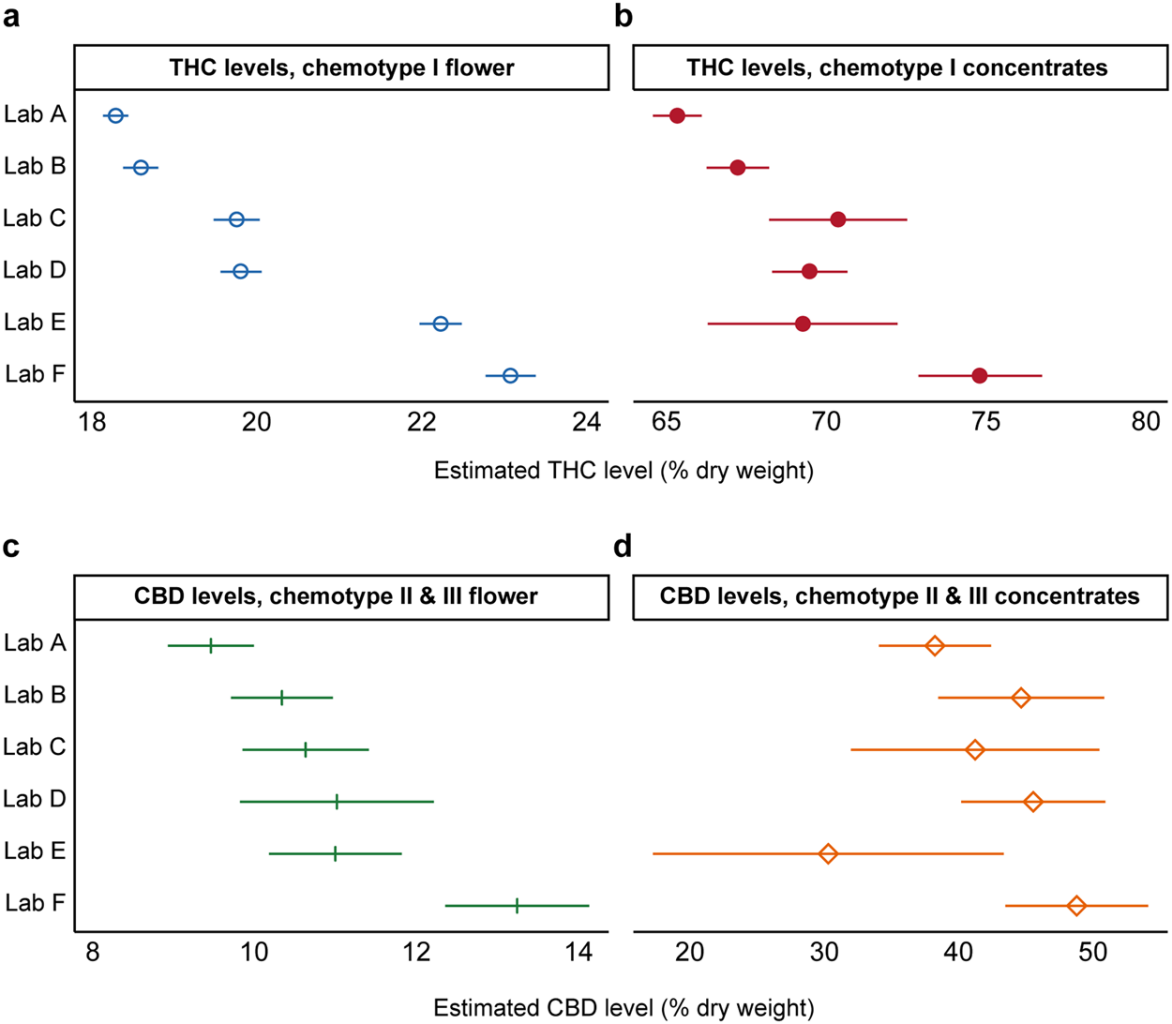
THC and CBD Levels Vary Between Labs After Controlling for Plausible Confounds. Average predicted values (+/− 99% confidence intervals) are shown, by lab, for (**a**) THC levels in chemotype I flower products (n = 161,933); (**b**) THC levels in chemotype I concentrate products (n = 33,888); (**c**) CBD levels in chemotype II and III flower products (n = 4,661); and (**d**) CBD levels in chemotype II and III concentrate products (n = 2,156) after adjusting for grower, strain-name, and time of measurement. Predicted values were generated from fixed-effects regressions with cluster-robust standard errors (see Methods).

To investigate potential determinants of interlab differences, we quantified the average cannabinoid levels reported by each lab after accounting for strain name, the producer-processor submitting samples for testing, and time of measurement (see Methods). Four separate regression models were estimated: (1) THC levels in chemotype I flower products (n = 161,933); (2) THC levels in chemotype I concentrate products (n = 33,888); (3) CBD levels in chemotype II and III flower products (n = 4,661); and (4) CBD levels in chemotype II and III concentrate products (n = 2,156). Large interlab variability in reported THC and CBD levels persisted across product categories after controlling for these factors (Fig. 3). Differences were observed for both flower (Fig. 3a,c) and concentrates (Fig. 3b,d). For chemotype I flower, the average adjusted total THC level for Peak Analytics (~23%) was significantly higher (p < 0.001; Wald test) than all other labs (Fig. 3a).

For chemotype I concentrates, Lab F’s average reported total THC (~75%) exceeded all other labs, including the lab reporting the second-highest average total THC (Lab E, ~70%). For total CBD levels in chemotype II and chemotype III flower, Lab F again reported the largest mean quantity, at about 13%, significantly higher (p < 0.01) than all other labs (Fig. 3c). For chemotype II and chemotype III concentrates, Lab F’s average products reported the highest CBD, but these estimates were uncertain due to the relatively small sample size (Fig. 3d). Overall, these results suggest that the observed differences between laboratories cannot be explained by differences in the producers, product types, or strain names of the samples being processed by each lab.

### Low-level cannabinoid measurements vary widely across laboratories

**Figure 4:**
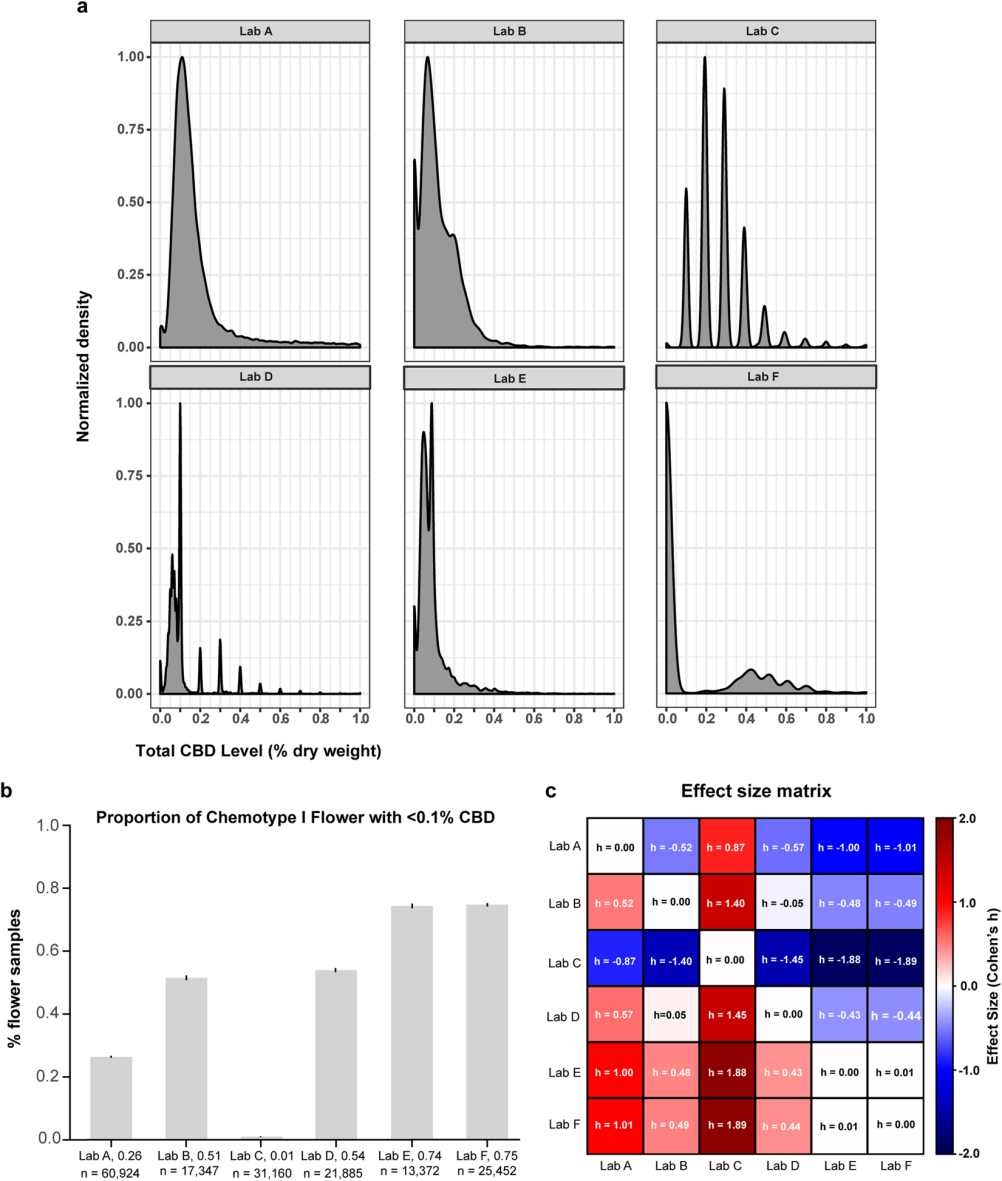
Labs differ in the propensity to detect low levels of CBD in chemotype I flower. (**a**) Kernel density plots of each lab’s distribution of total CBD levels below 1.0% dry weight for chemotype I flower (y-axis scaled to one). Most labs show a local maximum near 0.1% total CBD, which is a commonly reported LOQ. (**b**) Fraction of chemotype I flower with total CBD levels below 0.1% dry weight. Bars indicate proportions +/− 95% CI for a binomial proportion. (**c**) Effect size matrix indicating the magnitude of interlab differences shown in panel B. Effect size is quantified as Cohen’s h (see Methods).

Examination of THC:CBD distributions across laboratories indicated substantial variation in their propensity to report chemotype I strains with low total CBD levels (Figure S1, far right bins). To investigate this further, we plotted the density of chemotype I flower products with less than 1% CBD by dry weight (Fig. 4). The shape of these distributions varied somewhat across labs, likely due to methodological differences determining their Limit of Quantification (LOQ). Similar to what we observed in the THC:CBD histograms (Fig. 1b), these density plots indicate that differences exist between labs’ propensity to detect low-levels of CBD in chemotype I flower; they tend to display local maxima near 0.1%, which is the LOQ most labs report for cannabinoids.

To further quantify these differences, we compared the proportion of chemotype I flower having <0.1% total CBD across laboratories. There were dramatic differences between labs (Fig. 4b), with some reporting substantially more chemotype I flower with total CBD <0.1% than others. The volume of data caused even tiny interlab differences to reach high levels of statistical significance (p < 0.001 for all pairwise comparisons, except Lab E vs. Lab F, Mann-Whitney U test). Thus, we quantified the effect size of these differences by computing Cohen’s h for all pairwise comparisons (Fig. 4c; see Analytic Methods). Many of these interlab differences were of very large effect size (|h| >0.80, and often much greater), confirming that there are substantial differences in labs’ propensity to detect low levels of CBD in chemotype I flower. The analyses so far indicate that cannabinoid inflation and differences in the ability of labs to detect low-level cannabinoids both contribute to systematic differences in their reported measurements.

### Changes in THC Content of Commercial Cannabis Products Over Time

**Figure 5:**
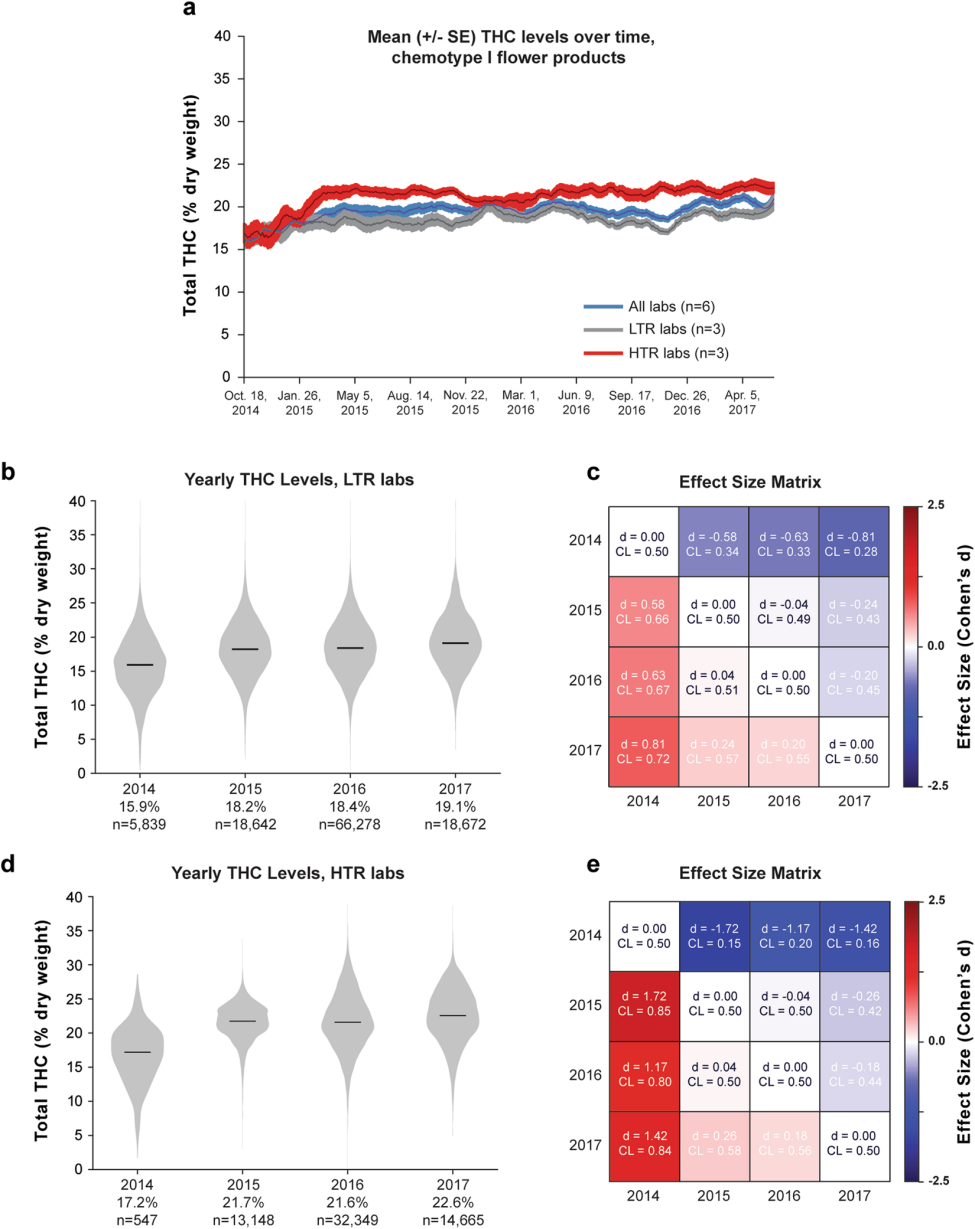
Mean THC Levels for Chemotype I Flower Products Over Time. (**a**) Total THC levels over time averaged across all labs or those reporting the highest or lowest mean THC levels. (**b**) Distribution of THC levels for each year on record for low THC reporting (LTR) labs. (**c**) Effect size matrix quantifying the mean difference in THC levels across years for LTR labs. (**d**) Distribution of THC levels for each year for high THC reporting (HTR) labs, and (**e**) the effect size matrix quantifying the magnitude of yearly differences.

While modern commercial strains contain higher THC levels than recreational cannabis from past decades^17,18^, it is unclear whether THC levels have continued climbing since Washington permitted adult-use cannabis. Thus, we looked for potential changes in the total THC content of commercial products in recent years. Because our previous analyses revealed systematic interlab variability in cannabinoid measurements, we sought to minimize the potential confound of lab-specific “cannabinoid inflation”. Thus, we quantified cannabinoid levels over time, separately for different subsets of laboratories: the three labs reporting the lowest mean THC levels (low THC reporting, LTR), the three reporting the highest mean THC levels (high THC reporting, HTR), as well as data pooled across laboratories. Figure 5a shows mean THC levels, averaged across labs, for chemotype I flower products from June 2014 through May 2017. While there was an upward trend from 2014 to early 2015, mean THC levels appear to have largely plateaued, with modest fluctuations since 2015 (Fig. 5a). This trend was evident in the pooled data as well as in LTR and HTR labs, although HTR labs showed a steeper increase in total THC levels from 2014 to 2015.

To further quantify changes in THC levels over time, we compared total THC levels for each year of data (Fig. 5b and c). Median THC levels for chemotype I flower rose from 2014 to 2015 but changed only slightly between 2015 and 2017. This was true whether we looked at the three LTR labs (Fig. 5b and c) or the three HTR labs (Fig. 5d and e). Again, large sample sizes allowed small differences in mean THC levels to reach statistical significance for all pairwise comparisons (p < 0.001, Mann-Whitney U test), except 2015 to 2016 for the HTR cohort (p = 0.334, Fig. 5d). After 2014, the effect sizes for year-to-year comparisons were small (Fig. 5b; |Cohen’s d| <0.23 for each comparison). Thus, we conclude that there has not been a substantial increase in the THC content of Washington state’s commercial cannabis flower from since 2015, although there were notable differences between LTR and HTR labs. For example, THC distributions from HTR labs were much more skewed (Fig. 5d; skew = −0.2) than for low-LTR labs (Fig. 5b; skew = 0.06). In addition, the increase in mean THC values from 2014 to 2015 was much larger for HTR than LTR labs (4.5% vs. 2.3%, respectively).

We were also interested in whether concentrates have increased in THC levels since 2014, as these products contain a much higher THC concentration. Mean THC levels across labs appeared to be relatively flat from 2014 to 2017 (Figure S3A). There was a small increase in THC levels from 2014 to 2015 for both cohorts of labs (Figure S3), although this was smaller than the increase observed for flower. From 2015 onward, there was a small decrease in mean THC levels for LTR and HTR labs (Figure S3). Thus, we conclude that, since 2015, there has not been a substantial increase in mean THC levels for commercial flower and concentrate products in Washington.

### THC Content Across Popular Commercial Categories: Indica, Sativa, and Hybrid

**Figure 6:**
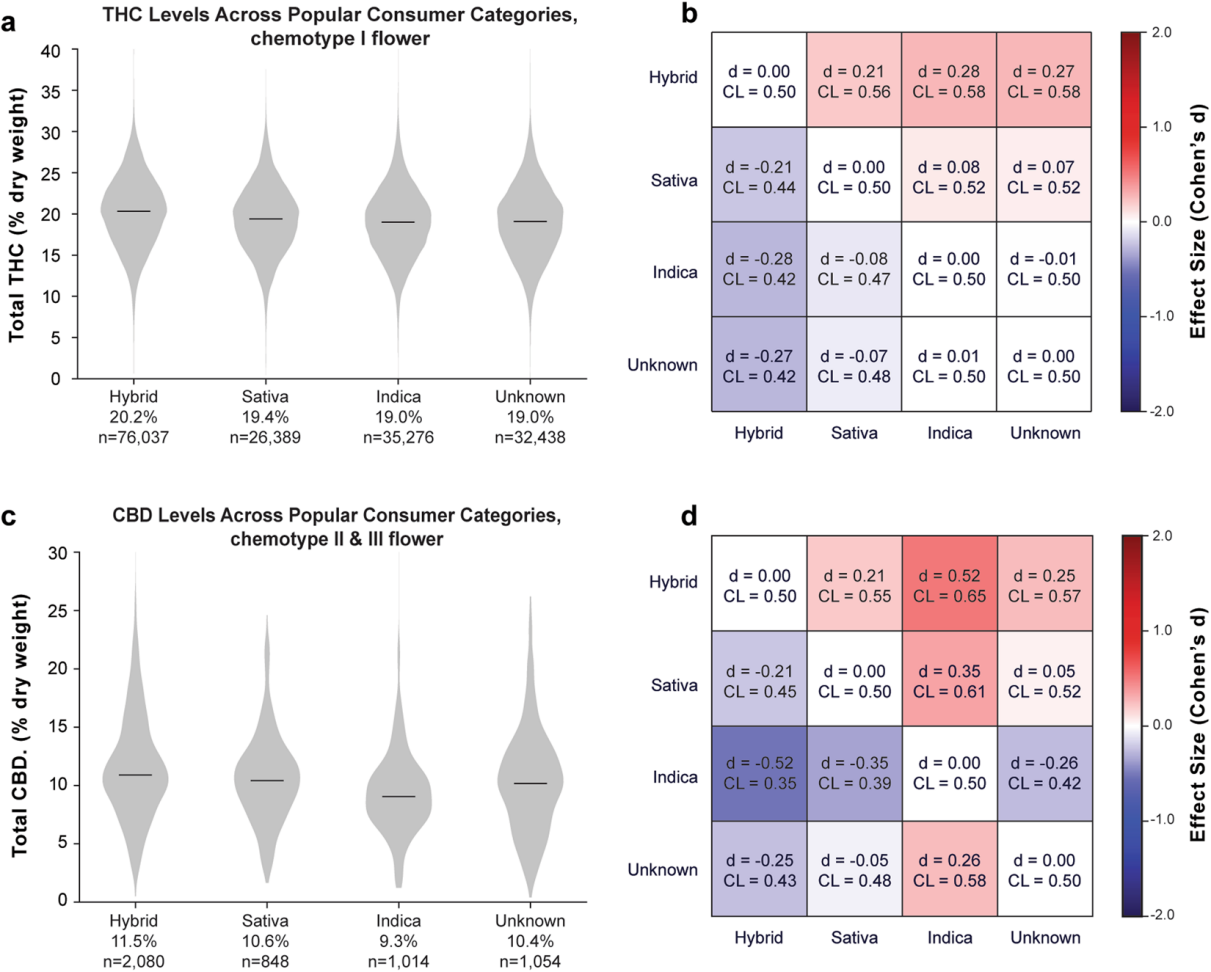
Total THC and CBD Levels Across Popular Consumer Strain Categories for Flower Products. (**a**) Distribution of THC levels across popular strain categories for chemotype I flower and (**b**) effect size matrix quantifying the magnitude of differences between them. (**c**) Distribution of CBD levels across the same categories for chemotype II and III flower and (**d**) effect size matrix quantifying the magnitude of differences between them.

The vernacular among cannabis users involves a triad of “indica,” “sativa”, and “hybrid” strains^19,20^. Recreational consumers and popular educational resources often attribute distinctive psychoactive effects to indica and sativa strains^21^, while scholars tend to be more skeptical of these claims^20,22^. In landraces, accessions from indica strains have been associated with more THC than sativas^13^ but indica and sativa recreational products sold in the Netherlands had similar THC content^14^. The term “strain,” although widely used, is not a botanically-accepted term for distinguishing plant varieties, and many scholars prefer the term “chemovar” in order to emphasize biochemical differences between specific *Cannabis* varieties^23^ (see Discussion). To investigate potential differences in cannabinoid content among commercial strain categories used by consumers, we looked at the distribution of THC content of indica, sativa, and hybrid flower samples in Washington’s commercial market. We matched test results using their producer-given strain name from the I-502 dataset to the Leafly.com strain database to retrieve their popular indica, sativa, or hybrid categorization (see Methods). This matching process yielded 166,594 flower results for analysis: 42,711 indica (25.6%), 31,822 sativa (19.1%), and 92,061 hybrid (55.3%) products. While hybrids had higher mean levels of THC compared to indicas and sativas, the distributions of THC content among indicas, sativas, and hybrids overlapped considerably (Fig. 6a–d).

To further quantify differences in THC content, we estimated a bivariate regression model of THC on strain category across all labs. The model indicates that hybrid strains have modestly greater THC content, on average, than either indica or sativa strains (Fig. 6e; hybrid vs indica: 1.22%, p < 0.001; hybrid vs sativa: 0.89%, p < 0.01). The difference in THC between sativa and indica could not be distinguished from sampling variability (sativa vs indica: 0.33%, p = 0.230). Moreover, the indica, sativa, hybrid distinction explained only a tiny fraction of THC variability between flower samples (r² = 0.016), and the differences in mean THC content had modest effect sizes (Hybrid vs Sativa: Cohen’s d = 0.283; Hybrid vs Indica: Cohen’s d = 0.206; Indica vs Sativa: Cohen’s d = −0.079). An analogous test for variability in CBD content across strain categories among chemotype 2 and chemotype 3 flower yielded similar results (Fig. 6f; hybrid vs indica: 2.17%, p < 0.01; hybrid vs sativa: 0.90%, p = 0.261; sativa vs indica: 1.26%, p < 0.05) with modest effect sizes (Hybrid vs Indica: Cohen’s *d = *0.148, Hybrid vs Sativa: Cohen’s *d = *0.068, Sativa vs Indica: Cohen’s *d = *0.268).

Importantly, the above results are sensitive to laboratory measurements. Data from most labs reflect the general pattern of hybrids having somewhat higher THC than indica and sativa, which are very similar. However, performing the same regression solely using flower products from Lab F (n = 22,049), which reports the highest mean THC levels, would not detect the higher average THC levels of hybrid flowers (hybrid vs indica: 0.47%, p = 0.21; hybrid vs sativa: 0.28%, p = 0.425). Repeating the analysis with solely flower products from Lab A (n = 50,610), the lab reporting the lowest mean THC levels, replicates the overall result, with hybrids having slightly higher THC than indica (1.13%, p < 0.001) and sativa (0.80%, p < 0.01).

### Cannabinoid Variation Within and Across Popular Commercial Strain Names

While commercial flower products fall into one of three chemotypes based on their THC:CBD ratio (Fig. 1), we wondered how much THC:CBD ratios varied within and between the popular commercial strain names that flower samples are given. Because the strain names of flower products submitted for laboratory testing are simply given by the producer-processor, they do not guarantee the true identity of the strain. In fact, personal correspondence with industry professionals indicated that we should expect flower samples submitted for testing to be mislabeled to some extent, perhaps due to business motives driving products to be given certain strain names based purely on their popularity and hence potential market value.

**Figure 7:**
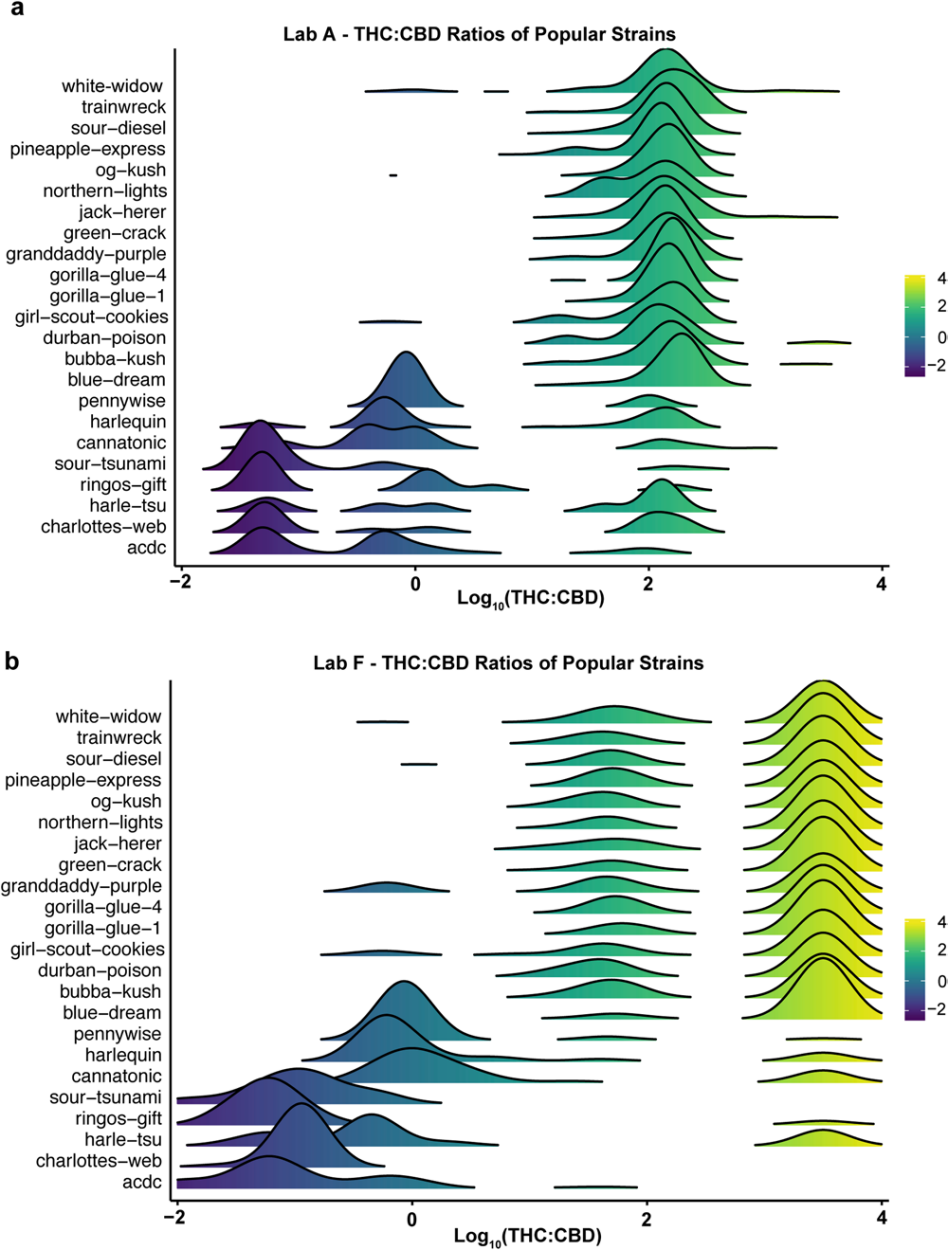
Distribution of THC-to-CBD Ratios Vary Across Popular Commercial Strain Names and Between Labs. THC-to-CBD ratios plotted on a logarithmic scale for cannabis flower samples across twenty-three popular commercial strain names for the single lab (Lab A) reporting the lowest (**a**) and the single lab (Lab F) reporting the highest (**b**) overall THC levels for cannabis flower.

To visualize differences between samples based on their popular commercial strain names, we plotted the THC:CBD ratio for 23 labeled strains (fifteen Chemotype I strains, three Chemotype II strains, and five Chemotype III strains) based on their consumer popularity (determined by cumulative pageviews on Leafly.com; see Methods) using data from the labs reporting the lowest (Fig. 7a; Lab A) and highest (Fig. 7b; Lab F) mean levels of THC across all flower products. This revealed clear differences in the THC:CBD profiles reported by each lab, as well as differences between the THC:CBD ratios of samples labeled with different strain names. Moreover, multi-modal distributions were apparent for many strains, with peaks sometimes at drastically different THC:CBD ratios. For example, “Charlotte’s Web” is a popular chemotype III strain that was specifically bred to have high total CBD and low total THC levels. Figure 7a clearly shows that many Charlotte’s Web flower samples tested in Washington commonly fit this profile, but many also fit the profile of chemotype II and even chemotype I products with high total THC levels. Thus, for subsequent analyses, we quantified data before and after filtering by modal chemotype. For example, for a strain name like Charlotte’s Web, this would mean considering only measurements within the chemotype III cutoffs (see Methods).

**Figure 8:**
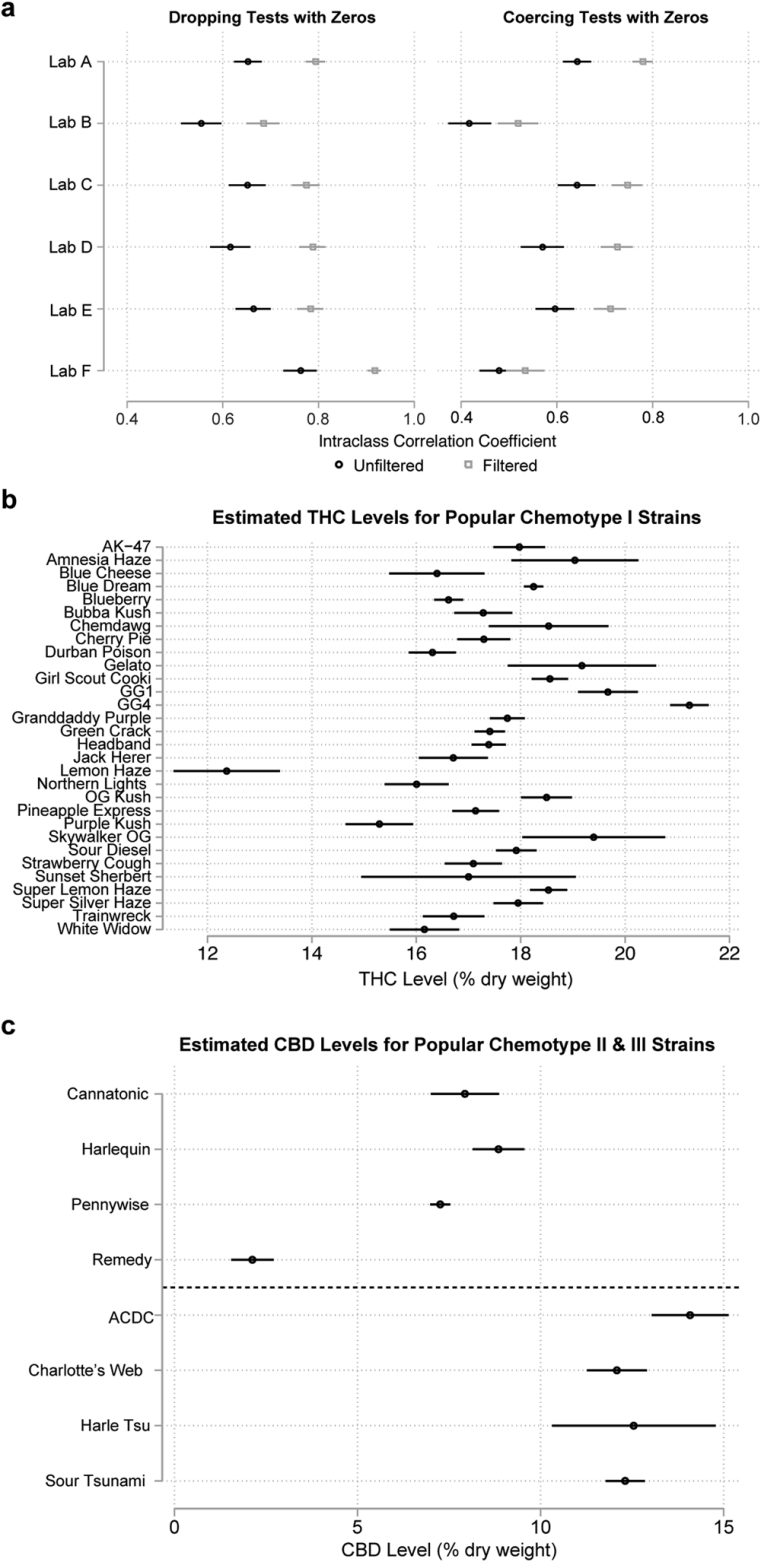
Popular Strain Names as Signal for THC and CBD Content. (**a**) Proportion of variation in log_10_ THC:CBD ratio explained by popular strain names (Intraclass Correlation Coefficient). 99% CIs are shown, by lab, before (black circles) and after (gray squares) filtering test results by the modal chemotype of each strain name. The ICC is shown both for dropping values for which 0% CBD or 0% THC is reported (left) and coercing cannabinoid ratios for these tests (see Methods). (**b**) Mean THC level of popular chemotype I strains. 99% CIs are shown after filtering by modal chemotype, for the lab reporting the lowest THC levels. (**c**) Mean CBD levels for popular chemotype II (above dotted line) and chemotype III (below dotted line) strain names. Results shown for the lab reporting the lowest mean THC levels.

To formalize how well colloquial strain names capture variation in THC:CBD profiles, we estimated a series of multilevel models with random intercepts for each strain^24^ to estimate the share of the total variation in the logged THC:CBD ratio explained by the strain name, before and after filtering by modal chemotype. This information is contained in the Intraclass Correlation Coefficient (ICC), a measure of similarity within-groups calculated as the ratio of the within-strain variance to the total variance (see Methods). The ICC is bounded by 0 and 1, where a value of 0 indicates that a sample’s strain name is completely uninformative of its THC:CBD ratio and a value of 1 indicates that a product’s strain name is perfectly predictive of its THC:CBD ratio. In these data, calculating the ICC is complicated by the relatively large number of test results with reported zero CBD (and thus an unbounded THC:CBD ratio). Thus, ICCs pre-and-post filtering are shown both after omitting results with 0 CBD and coercing these results to tail values (Fig. 8a; see Methods). When using tail values, results with zero reported CBD were coerced to a ratio of 3.5 and results with zero THC coerced to −2.0. These values correspond approximately to the most extreme values observed in the data (see Fig. 1b).

Across all labs, the pre-filtered strain ICC was 0.57 (omitting results with zero reported CBD or THC) or 0.41 (coercing results with zero reported CBD to 3.5 and zero THC to −2.0). After filtering, the ICCs increase, respectively, to about 0.71 and 0.51. This overall test conceals significant variation between labs. Figure 8a shows the ICCs with 99% Confidence Intervals, by lab, before and after strain name filtering, separately for the two methods of handling results with reported zero CBD. In general, a large portion of the variation in THC:CBD ratio was attributable to strain name. This quantity varied substantially between labs, and filtering results outside a strain’s modal chemotype typically boosted the proportion of variation explained by strain by between about 0.10 and 0.15, depending on the model. In all but one case—Lab F, with missing CBD results coerced to a ratio of 3.5—filtering resulted in a statistically significant (p < 0.01, z test) increase in the proportion of variation explained by strain. Labs varied substantially in the proportion of zero CBD test results, and, consequently, the ICC was more sensitive to the handling of missing data for those labs. For Lab F, the lab with the highest proportion of zero CBD results, the ICC was very sensitive to the handling of missing data; the ICC was 0.76 and 0.48, respectively, before filtering, and 0.92 and 0.53, after filtering. In contrast, Lab A, which had few zero CBD values, had a stable ICC across both methods, with pre-filtered ICCs of 0.65 and 0.64, respectively, and post-filtering ICCs of 0.79 and 0.78.

Given these observations, we compared total THC and CBD levels across strains only after filtering data by laboratory and modal chemotype. Using data from only one laboratory ensured that the same laboratory testing protocol was used to measure cannabinoids across flower samples. We chose to use data from Lab A, which had the largest number of flower samples. Comparing mean THC or CBD levels revealed clear and statistically significant differences (p < 0.01, Wald Test) between many strain names, even within chemotypes (Fig. 8). These results suggest that strain names can provide meaningful, though variable, signals of the composition of flower samples. Filtering results to the strain’s modal chemotype boosted the proportion of variation explained by strain clustering by about 0.15. Furthermore, the strength of this signal varies between labs, particularly with respect to the CBD content of high-THC flower.

## Discussion

Our results confirm that commercial cannabis strains in Washington fall into three principal chemotypes defined by their THC:CBD ratio, similar to landrace^13^ and commercial Dutch strains^14^. While this result is unsurprising given the biological constraints on cannabinoid production^25,26^, we were able to use this dataset to investigate outstanding questions about commercial cannabis products widely used by consumers today. These included formal analysis of discrepancies in the cannabinoid levels reported by different laboratories, trends in THC content over time, and systematic differences in the THC:CBD profiles between flower samples with different labeled strain names.

A key area of concern for legal cannabis consumers, industry professionals, and state regulators is the accuracy of the state-mandated testing data that is required to be displayed on product packaging. Recent media reports of “THC inflation” and high-profile suspensions of state-licensed testing facilities have prompted concern over the accuracy of the cannabinoid content of legal cannabis products^27^. Our analyses revealed clear, systematic differences in the results obtained by different testing facilities in Washington, with some labs consistently reporting higher or lower levels of cannabinoids than others. Moreover, these differences could not be explained by differences in the producer or strain name associated with the samples being submitted, suggesting that discrepancies between labs are likely caused by systematic differences in their testing methodologies. It is crucial that precise standards are adopted by the industry to ensure that laboratories produce results that are reproducible across labs, independent of the exact testing method used, with the ultimate goal of reporting results that consumers can trust. Our analyses provide a potential framework for quantitatively evaluating laboratory quality, including labs’ sensitivity to detecting low-level cannabinoids (Fig. 4), agreement with independent measurements of cannabis products in scholarly journals (discussed below), and differences between labs after accounting for other characteristics.

In our analyses of Washington labs, median THC levels for chemotype I flower varied considerably (Fig. 2), ranging from 17.7% to 23.2%. The results reported by labs with lower median THC levels are in better agreement with independent measurements of cannabis flower from legal markets^8,28,29^. For example, Vergara *et al*. (2017) reported total THC levels for commercial flower samples in several U.S. cities, finding that flower samples averaged approximately 19% total THC in Seattle, WA, with even lower levels (~15%) reported for Denver, CO, Sacramento, CA, and Oakland, CA^8^. Other measurements of flower samples originating from medical cannabis patients in California found median total THC levels for chemotype I samples to be approximately 17% dry weight^28^. Another study measured THCA across a variety of popular cultivars and found mean THCA levels to be just over 16% dry weight for specimens with a broad-leaflet phenotype vs. 14% for those with a narrow leaflet phenotype (total THC levels were not reported)^29^.

Reliable cannabis laboratory testing is an attainable goal. In the absence of federal regulations in the United States for the foreseeable future, it will be incumbent on state regulators to implement universal testing standards for cannabis laboratories. But states have extensive experience in this arena, regulating laboratories that analyze drinking water and evidence from crime scenes. They need only hold cannabis laboratories to similar standards. A first step may be to require that cannabis labs, like other testing facilities, receive third-party accreditation of compliance with International Organization for Standardization (ISO) 17025 guidelines for testing and calibration laboratories by auditors who are themselves ISO-accredited^30^. Second, states could require that cannabis labs adhere to a standardized testing protocol for analyzing cannabis to ensure consistency between labs. Such protocols have already been developed. For example, the American Herbal Pharmacopoeia published a 64-page monograph called Cannabis Inflorescence detailing analytical procedures for cannabinoid detection and quantification to establish benchmark methods^31^. The American Herbal Products Association’s points readers to Cannabis Inflorescence for guidance regarding “specific analytical methods” in its recommendations for regulators^32^. Finally, states should implement regular “round-robin” audits, sending a blinded, common sample for testing across various laboratories.

Overall, our results are consistent with reports of “cannabinoid inflation” by certain laboratories^10^ and point to intrinsic characteristics of labs as driving the observed variability, rather than confounds introduced from different producers, product types, or strain names being processed by each lab. Previous analysis of data from Washington’s traceability dataset found a statistically significant relationship between the price per gram of flower and both total THC and total CBD levels^33^, which may provide an economic incentive for cannabis producers to seek test results with higher total THC or CBD levels.

Restricted to the lab reporting the lowest THC levels for chemotype I flower (median = 17.7%), the 99th percentile THC level is 27.0%, compared to 31.8% for the lab reporting the highest THC levels. This suggests that flower samples with total THC levels approaching or exceeding 30% are very rare, and that many flower products labeled as containing >30% total THC by dry weight may be inaccurate. Our analysis of changes in THC content over time suggest that THC levels have not risen substantially, at least since 2015 (Fig. 5). While it is well-known that modern recreational cannabis contains higher levels of THC than it did in previous decades^17^, this apparent plateauing in recent years may be expected due to biological limits imposed on cannabinoid production. In *Cannabis*, total potential THC levels are determined by levels of THCA synthesized within the plant, which is under genetic control^26,34^.

In general, our results do not suggest that flower samples labeled as indica, sativa, and hybrid differ substantially in terms of total THC content or THC:CBD profiles, as the indica/sativa/hybrid typology accounted for only about 1% of the relative variability in THC content (r² = 0.016). Samples labeled sativa vs. indica in Washington’s commercial market do not differ in THC content, similar to what was documented among commercial strains in the Netherlands^14^. However, we did find that hybrid strains have slightly higher levels of total THC. Since hybrid strains are produced by crossing indica and sativa varieties, this result is somewhat counterintuitive. We interpret it as potentially owing to the selective breeding of hybrid strains to maximize THC content for commercial purposes. It is also possible that, on average, these group are grown under different growing conditions, such as indoors vs. outdoors.

While there were no substantial differences in the THC or CBD content between flower samples labeled as indica vs. sativa, our analyses were limited by the contents of the Washington traceability dataset, which did not include measurements of other phytocannabinoids or terpenes, a major class of aromatic compounds produced by *Cannabis* that likely modulate the effects of phytocannabinoids^7,23,25,35^. It remains possible that indica and sativa samples differ systematically in their full phytocannabinoid or terpene profile. Indeed, a recent analysis of cannabinoid and terpene profiles from Dutch flower samples found several terpenes that may serve as markers for indica- vs. sativa-type samples^14^. Other recent work^23^ has shown how more complete sets of biochemical measurements, including terpenoid profiles, can be used to define multiple distinct *Cannabis* chemovars.

Since flower samples with different strain names within each of the three principal chemotypes can be differentiated somewhat by their THC:CBD ratio, more detailed analysis of the biochemical composition of commercial strains may uncover further differences. Indeed, previous work has attempted to differentiate strains based on analysis of more complete cannabinoid and terpene profiles^14,28,36,37^ in an effort to define *Cannabis* “chemovars”^23,38^. An important area of future research will be to define the chemovars for commercially available cannabis products in legal U.S. markets. Applying computational approaches to large-scale cannabinoid and terpene datasets will allow cannabis strains and products to be organized according to their biochemical constituents rather than colloquial strain names. Accurate data quantifying the principal constituents of cannabis could foster the development of “product maps” that organize commercial products based on levels of their psychoactive and medically-relevant compounds. However, doing so requires having accurate lab testing data in the first place, which requires the establishment of laboratory testing standards that ensure the correct identification and quantification of the contents of cannabis products. Our results highlight the need for such standards given the large, systematic differences observed between laboratories in Washington.

## Methods

### Data

#### Washington I-502 Cannabis Test Data

We submitted public records requests for all test results registered with the Washington State Liquor and Cannabis Board (LCB), the agency which regulates cannabis sales. The request was made on August 7, 2017 and the LCB provided us the raw data files on August 14, 2017. These data contained cannabinoid profiles, grower and laboratory information, and test date for 304,123 test results from June 2014 through May 2017 (excluding a very small number of test results that were dropped from the dataset for containing mathematically or biologically impossible values, such as THC content greater than 100%). The data files from which we extracted data for our analyses are attached online.

We focused our analyses on two product categories: flower and concentrates. “Flower” refers to products refer to those comprised of the mature female flower of the cannabis plant, which most cannabis products are derived from. Our analyses of flower products came from data in the “Flower Lot” group, which can be found in the InventoryLabel column of the I-502 dataset accompanying this paper. Our analyses of concentrate products refer to data with the “Hydrocarbon Wax” InventoryLabel, which was the largest single group for product types that would normally be considered concentrates. We did not explore data from other product categories in this study, including “Hash,” “Marijuana Infused Edibles,” and a variety of others. We encourage other scholars to mine this dataset for further insights.

#### Calculation of THC and CBD Levels

*Cannabis* plants do not synthesize THC or CBD; instead, they synthesize the cannabinoid acids THCA and CBDA, which are made from a common precursor^12^ and must be decarboxylated (e.g. by heat energy) to yield the phytocannabinoids THC and CBD. Thus, cannabis products, especially flower samples, contain mainly THCA and/or CBDA, as well as small levels of THC and CBD that result from spontaneous decarboxylation during the cultivation process. Total THC or CBD levels, in units of percent of dry weight, are typically calculated as:

1 $${Total}\,{CBD}=(0.877\times {CBDA})+{CBD}$$

2 $$Total\,THC=(0.877\times THCA)+THC$$

Where THCA and THC refer to the percent dry weight concentration of each cannabinoid present in a cannabis product, and 0.877 is the scaling factor accounting for the difference in molecular weight between THCA and THC. “Total THC” or “Total CBD” refers to this value, which is the maximum potential THC or CBD content of a cannabis product, assuming 100% decarboxylation of cannabinoid acids. This is the standard way of reporting “total” cannabinoid levels for legal cannabis products in Washington. Unless otherwise noted, “THC levels” or “total THC levels” refers to this value (similar for CBD levels).

#### Leafly data

Leafly’s database of strains was used to group test results, as described in the matching process below. The Indica, Sativa, or Hybrid categorization from Leafly.com allowed us to categorize strains according to their popular consumer designations. Our data matched to 1,318 unique commercial strains. The popularity of consumer strain names were determined by cumulative pageviews of the strain pages on Leafly.com.

### Analytic Methods

#### Matching Strain Names

Strain names in the Washington I-502 seed-to-sale traceability data were matched to a database of Leafly strains to retrieve information about strain popularity and indica/sativa categorization. The matching process had two stages. First, I-502 data strain names were standardized using regular expressions to delete reference to grower’s names and cities, correct spelling irregularities, and expand acronyms (e.g. “GDP” referring to the strain “Granddaddy Purple”). Second, the processed I-502 strain names were matched to the Leafly database using open-source implementations of two different Approximate String Matching algorithms (Ratcliff/Obershelp and Levenshtein Distance). If the two algorithms agreed, then the test result was assigned the Leafly strain. Through this process, used to minimize the number of false positive matches, 214,747 (70.6%) results were matched to Leafly strains, including 166,594 for flower samples and the remainder for other product types. A complete list of raw strain names and their corresponding Leafly match can be found in the Supplementary Information files.

#### Chemotype Cutoffs

Following previous scholars^13^, test results were classified into chemotypes based on the distinct groups visible after plotting the log_10_ THC-to-CBD ratio. For our analyses, we defined chemotype I strains as any with a 5:1 THC:CBD ratio or greater, chemotype III strains as any with a 1:5 THC:CBD ratio or lower, and the remainder of products were classified as Chemotype II. We called the “strain chemotype” the modal chemotype among test results of that strain (in the matched Leafly data).

#### Statistical Significance and Effect Size for Very Large Samples

Due to the size of the I-502 dataset we analyzed, statistical comparisons often involved samples with tens of thousands of data points each, and statistically significant results were often achieved even for trivial differences between sample means with highly overlapping distributions. Thus, we calculated effect size in a variety of ways to determine whether differences between samples were relatively large or small. When comparing continuous distributions, we computed effect size in two ways: Cohen’s d^15,39^ and a “Common Language” (CL) effect size metric^16^.

The CL effect size measures the probability that a randomly selected value from one population will be greater than a randomly selected value from another while Cohen’s d measures effect size as the difference between two samples means divided by their pooled standard deviation^15^:

3 $$d=\,\frac{{y}_{i}-{y}_{j}}{\sqrt{\frac{({n}_{i}-1){s}_{i}^{2}+({n}_{j}-1){s}_{j}^{2}}{{n}_{i}+{n}_{j}-2}}}$$

where *y* denotes each sample mean, and *n* and *s*^2^ are the size and variance of each sample, respectively.

For example, Fig. 2a shows that Lab A (Confidence Analytics) and Lab B (Analytical 360) report slightly different median THC values for flower (17.6% and 18.3%, respectively) and display highly overlapping distributions. Performing a t-test to measure a difference in the means (17.5% and 18.0%, respectively) returns p-value < 1.1 × 10^−52^. While the t-test indicates a statistically significant result, |Cohen’s d| = 0.13, indicating a “small effect”^15^. Similarly, the CL effect size is 0.46, indicating that a random value from Confidence Analytics will be greater than a random value from Analytical 360 46% of the time, which is close to what identical distributions would display (50%). The effect size matrices displayed in this paper allow one to assess whether differences between samples are relatively large or small, which is especially useful when large sample sizes allow small differences to reach statistical significance.

For comparing different proportions, we computed Cohen’s h^15^, which is the difference between two proportions’ arcsine transformation:

4 $$h=2arcsin\sqrt{{p}_{i}}-2arcsin\sqrt{{p}_{j}}$$

#### Cannabinoid Inflation Regression Models

Each of the four regression models shown employed a fixed-effect transformation for each grower-strain combination to absorb heterogeneity in cannabinoid content attributable to these factors^40^. Written in the Least Squares Dummy Variable formulation, each equation takes the form:

5 $${y}_{tls}={\beta }_{1}Dat{e}_{t}+\sum _{s=1}^{S}{\delta }_{s}{D}_{ts}+\sum _{l=1}^{L}{\gamma }_{l}{D}_{tl}+{\eta }_{tls}$$

where *y* denotes the cannabinoid content of a particular test *t* from lab *l* of producer-strain *s*, *S* indexes each producer-strain combination (omitting one), and *L* indexes each lab (omitting one); *D*’s are indicator variables denoting group (lab and strain) membership, and η is the idiosyncratic model error. The primary quantity of interest is *γ*, the expected deviation in cannabinoid content for each lab relative to the omitted lab, adjusting for grower/strain and test date. As a robustness check, for regressions subsetted to concentrate products, an additional dummy variable for type of concentrate (e.g. “Bubble Hash”, “Butane Hash Oil”, “CO2 Hash Oil”) was included to control for potential heterogeneity between labs in their stock of concentrate products, since concentrates differ in potency by their production method^41^; inclusion of this variable does not materially alter the results. Standard errors were clustered by grower-strain, since the number of clusters is large (there were 22,716 grower-strain combinations) and residuals are likely to be correlated within clusters^42^. Average covariate-adjusted cannabinoid values across labs are plotted with 99% Confidence Intervals estimated through the delta-method using Stata’s *margins* command, holding covariates at their actual values^43^.

#### THC and CBD Variation Across Popular Commercial Strains

To examine clustering in THC:CBD ratio by strain name, we estimated unconditional two-level hierarchical models with random intercepts for each strain, across all labs and separately by lab. Each specification took the following form:

6 $${r}_{ts}=\gamma +{\mu }_{s}^{(2)}+{\varepsilon }_{ts}$$

where *r* is the THC:CBD ratio for each test *t* of strain *s*, *γ* is the “global-mean” THC:CBD ratio across all strain names, *μ* is the level-two random intercept for each strain (capturing the variance of the mean for each strain around the overall mean), and *ε* is the deviation of each test from its strain mean. The Intraclass Correlation Coefficient (ICC) is calculated as the ratio of the within-group variance to the total variance, which can be decomposed into the within-group variance and the between group variance. Here, the ICC *ρ* is the ratio of the within-strain variance to the sum of the between-strain variance and within-strain variance:

7 $$\rho =\frac{{\sigma }_{w}^{2}}{{\sigma }^{2}}=\frac{{\sigma }_{w}^{2}}{{\sigma }_{w}^{2}+{\sigma }_{b}^{2}}$$

which is strictly bounded between 0 (when there is zero within-strain variance) and 1 (when there is zero between-strain variance). The random intercept models were estimated using Stata’s *mixed* command; the post-estimation command *estat icc, l(99)* was used to calculate 99% Confidence Intervals with a logit transformation to accommodate the restricted domain of the ICC and the delta method to estimate standard errors.

#### Strain Category Cannabinoid Estimation

Regression was used to estimate whether indica, sativa, and hybrid flower products differed systematically in THC and CBD. Standard errors were clustered by strain, since indica, sativa, hybrid is a strain-level property. There were 1,318 unique strains.

#### Data Availability Statement

All data generated or analyzed during this study are included in this published article (and its Supplementary Information files). They are also available in the Harvard Data Repository (10.7910/DVN/E8TQSD).

## Electronic supplementary material

Supplemental Figures

Supplementary Dataset 1

## References

[1] Grinspoon, L. History of Cannabis as a Medicine. MAPS http://www.maps.org/research-archive/mmj/grinspoon_history_cannabis_medicine.pdf (2005).

[2] Whiting PF (2015). Cannabinoids for medical use: A systematic review and meta-analysis. JAMA..

[3] National Academies of Sciences, Engineering, and Medicine. The health effects of cannabis and cannabinoids: The current state of evidence and recommendations for research. National Academies Press. 10.17226/24625 (2017). 28182367

[4] Geiger, A. Support for marijuana legalization continues to rise. Pew Research Center http://www.pewresearch.org/fact-tank/2016/10/12/support-for-marijuana-legalization-continues-to-rise Accessed September **29**, 2017 (2016).

[5] Compton WM, Han B, Hughes A, Jones CM, Blanco C (2017). Use of marijuana for medical purposes among adults in the United States. JAMA..

[6] Barry R. A. & Glantz S. A public health framework for legalized retail marijuana based on the US experience: Avoiding a new tobacco industry. *PLoS Medicine*. **13**(9) (2016). 10.1371/journal.pmed.1002131 PMC5038957 27676176

[7] Andre, C. M., Hausman, J. F. & Guerriero, G. Cannabis sativa: The plant of the thousand and one molecules. *Frontiers in plant science*. **7**(19) (2016). 10.3389/fpls.2016.00019 PMC4740396 26870049

[8] Vergara D (2017). Compromised external validity: Federally produced Cannabis does not reflect legal markets. Sci Rep..

[9] Morgan CJ, Schafer G, Freeman TP, Curran HV (2010). Impact of cannabidiol on the acute memory and psychotomimetic effects of smoked cannabis: naturalistic study [corrected]. Br J Psychiatry..

[10] Coughlin-Bogue T. Leafly Investigation: Is Washington’s Top Cannabis Lab Inflating THC Numbers? *Leafly*. https://www.leafly.com/news/industry/leafly-investigation-washingtons-top-cannabis-lab-inflating-thc-numbers Accessed September **13**, 2017 (2017).

[11] Hillig KW (2004). Genetic Evidence for Speciation in Cannabis (Cannabaceae). Genetic Resources and Crop Evolution..

[12] De meijer (2003). The inheritance of chemical phenotype in Cannabis sativa L. Genetics..

[13] Hillig KW, Mahlberg PG (2004). A chemotaxonomic analysis of cannabinoid variation in Cannabis (Cannabaceae). American Journal of Botany..

[14] Hazekamp A, Tejkalová K, Papadimitriou S (2016). Cannabis: from cultivar to chemovar II—a metabolomics approach to Cannabis classification. Cannabis and Cannabinoid Research..

[15] Cohen, J. *Statistical Power Analysis for the Behavioral Sciences*. (Routledge, 1988).

[16] McGraw KO, Wong SP (1992). A common language effect size statistic. Psychological bulletin..

[17] ElSohly MA (2016). Changes in cannabis potency over the last 2 decades (1995–2014): Analysis of current data in the United States. Biological Psychiatry.

[18] Mehmedic Z (2010). Potency trends of Δ9-THC and other cannabinoids in confiscated cannabis preparations from 1993 to 2008. J Forensic Sci..

[19] Cervantes J. *The Cannabis Encyclopedia: The Definitive Guide to Cultivation & Consumption of Medical Marijuana*. (Van Patten Publishing, 2015).

[20] McPartland, J. M. Cannabis sativa and Cannabis indica versus “sativa” and “indica” in *Cannabis sativa L.-Botany and Biotechnology* (eds. Chandra S., Lata H., ElSohly M.) 101–121 (Springer, Cham, 2017).

[21] Pearce DD, Mitsouras K, Irizarry KJ (2014). Discriminating the effects of Cannabis sativa and Cannabis indica: a web survey of medical Cannabis users. The Journal of Alternative and Complementary Medicine..

[22] Piomelli D, Russo EB (2016). The Cannabis sativa versus Cannabis indica debate: An interview with Ethan Russo, MD. Cannabis Cannabinoid Res..

[23] Lewis, M. A., Russo, E. B. & Smith, K. M. Pharmacological Foundations of Cannabis Chemovars. *Planta Medica* (2017). 10.1055/s-0043-122240 29161743

[24] Gelman, A. & Hill, J. *Data Analysis Using Regression and Multilevel Hierarchical Models*. (Cambridge University Press, 2007).

[25] Russo EthanB (2011). Taming THC: potential cannabis synergy and phytocannabinoid‐terpenoid entourage effects. British journal of pharmacology.

[26] De Meijer E. P. The chemical phenotypes (chemotypes) of Cannabis in *Handbook of Cannabi*s (ed. Pertwee, R) 89–110 (Oxford University Press, 2014).

[27] Coughlin-Bogue T. Washington Labs Launch Effort to Address CredibilityCrisis. *Leafl*y. https://www.leafly.com/news/industry/washington-labs-launch-effort-to-address-credibility-crisis Accessed September **16**, 2017 (2017).

[28] Elzinga, S, Fischedick, E. S., Podkolinski, R. & Raber, J. C. Cannabinoids and Terpenes as chemotaxonomic markers in Cannabis. Natural Products Chemistry & Research. **3**(4) (2015).

[29] Lynch RC (2016). Genomic and Chemical Diversity in Cannabis. Critical Reviews in Plant Sciences.

[30] Unger, P., Brauninger, R., Hudalla, C., Holmes, M. & Sherman, B. Standards for Cannabis Testing Laboratories. Eugene, OR: Cannabis Safety Institute. *Retrieved* **4**(01) (2014).

[31] Upton, R. & ElSohly, M. (Eds.). Cannabis Inflorescence: Cannabis Spp.; Standards of Identity, Analysis, and QualityControl. *American Herbal Pharmacopoeia* (2013).

[32] Cannabis Committee of the American Herbal Products Association. Recommendations for regulators—Cannabis operations. Available from: http://www.ahpa.org/Portals/0/Documents/AHPA_Recommendations_for_Regulators_Cannabis.

[33] Smart R, Caulkins JP, Kilmer B, Davenport S, Midgette G (2017). Variation in cannabis potency and prices in a newly legal market: evidence from 30 million cannabis sales in Washington state. Addiction..

[34] Weiblen GD (2015). Gene duplication and divergence affecting drug content in Cannabis sativa. New Phytol..

[35] McPartland JM, Russo EB (2001). Cannabis and cannabis extracts: Greater than the sum of their parts?. Journal of Cannabis Therapeutics.

[36] Aizpurua-Olaizola O (2016). Evolution of the cannabinoid and terpene content during the growth of Cannabis sativa plants from different chemotypes. J. of Natl Products..

[37] Casano S, Grassi G, Martini V, Michelozzi M (2011). Variations in terpene profiles of different strains of Cannabis sativa L. Acta Hortic..

[38] Hazekamp A, Fischedick JT (2012). Cannabis - from cultivar to chemovar. Drug Test Anal..

[39] Sawilowsky S (2009). New effect size rules of thumb. Journal of Modern Applied Statistical Methods..

[40] Wooldridge, J. M. *Introductory Econometrics: A Modern Approach*. pp. 484–485 (Nelson Education, 2015).

[41] Raber JC, Elzinga S, Kaplan C (2015). Understanding dabs: Contamination concerns of cannabis concentrates and cannabinoid transfer during the act of dabbing. The Journal of Toxicological Sciences..

[42] Cameron AC, Miller DL (2015). A practitioner’s guide to cluster-robust inference. Journal of Human Resources.

[43] Williams, R. Using the margins command to estimate and interpret adjusted predictions and marginal effects. *Stata Journal*. **12**(2), 308 (2012).

