# Author Correction: The Cannabinoid Content of Legal Cannabis in Washington State Varies Systematically Across Testing Facilities and Popular Consumer Products

**DOI:** 10.1038/s41598-020-69680-x

**Published:** 2020-08-27

**Authors:** Nick Jikomes, Michael Zoorob

**Affiliations:** 1 Division of Data Science, Leafly Holdings, Inc., Seattle, WA 98104 USA; 2 Department of Government, Harvard University, Cambridge, MA 02138 USA

Correction to: *Scientific Reports* 10.1038/s41598-018-22755-2, published online 14 March 2018

The original version of this Article contained an open access replication dataset containing the data used for our analyses. This dataset contained duplicate entries which arose from merging two samples of the I-502 database spanning two distinct, but overlapping, time frames, obtained through public records request. One subset of the database comprised tests over the period January 2016-May 2017; a second subset contained tests from June 2014-April 2017. To account for the overlapping time periods of the I-502 data, we used custom Python code to remove duplicate entries. However, our data deduplication code did not work as expected and marked as duplicate only some, but not all, of the rows in the merged dataset. Removal of these duplicates and re-running our analyses did not substantively alter any results or conclusions. Figures, sample sizes, and numerical results have been updated to reflect the revised full dataset of 215,285 testing results.

In the “THC and CBD Measurements Vary Widely Across Testing Laboratories” section,

**Figure 1:**
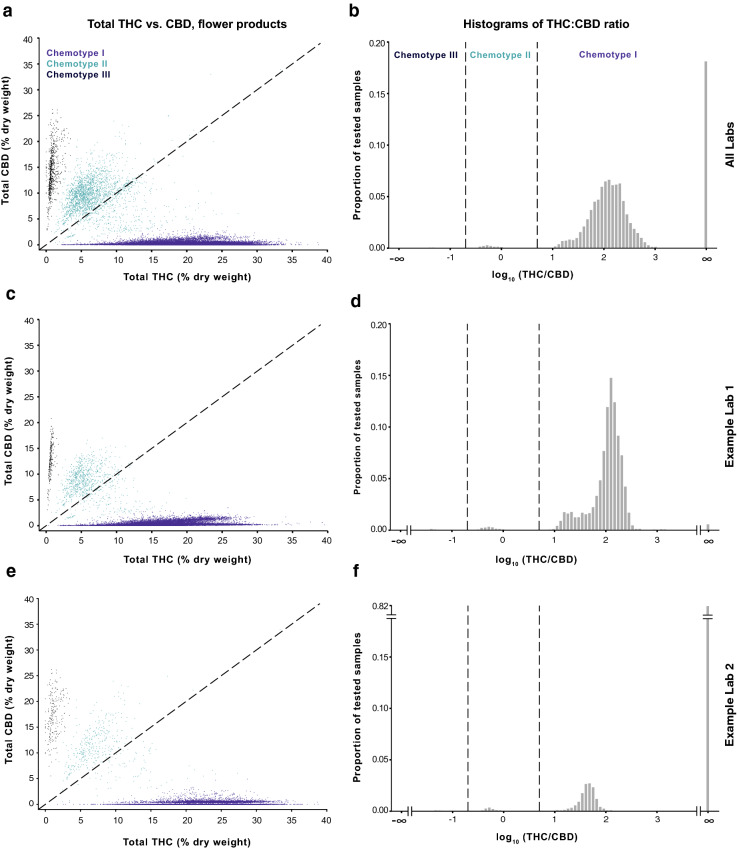
The THC:CBD ratio defines three broad chemotypes of commercial cannabis flower measured by testing labs in Washington. Left column: Scatterplots of total THC vs. total CBD levels for cannabis flower. Right column: Histograms showing the THC:CBD ratio on a log scale and indicating the proportion of flower samples for each chemotype. Data are displayed for measurements batched across all Labs A-F (panels a-b; n = 123,977), for the lab reporting the lowest mean total THC levels (Lab A; panels c-d; n = 41,189), and the lab reporting the highest mean total THC levels (Lab F; panels e–f; n = 23,133). Histograms for each of the six labs contributing to batched data in panels a-b are shown in Figure S1. Panels a and c were subsampled to n = 50,000 for visualization purposes.

“For example, the median total THC content for chemotype I flower products ranged from 17.7% to 23.2% between the labs reporting the lowest and highest THC levels, respectively (Fig. 2a; labs A-F ordered from lowest to highest median reported THC levels).”

**Figure 2:**
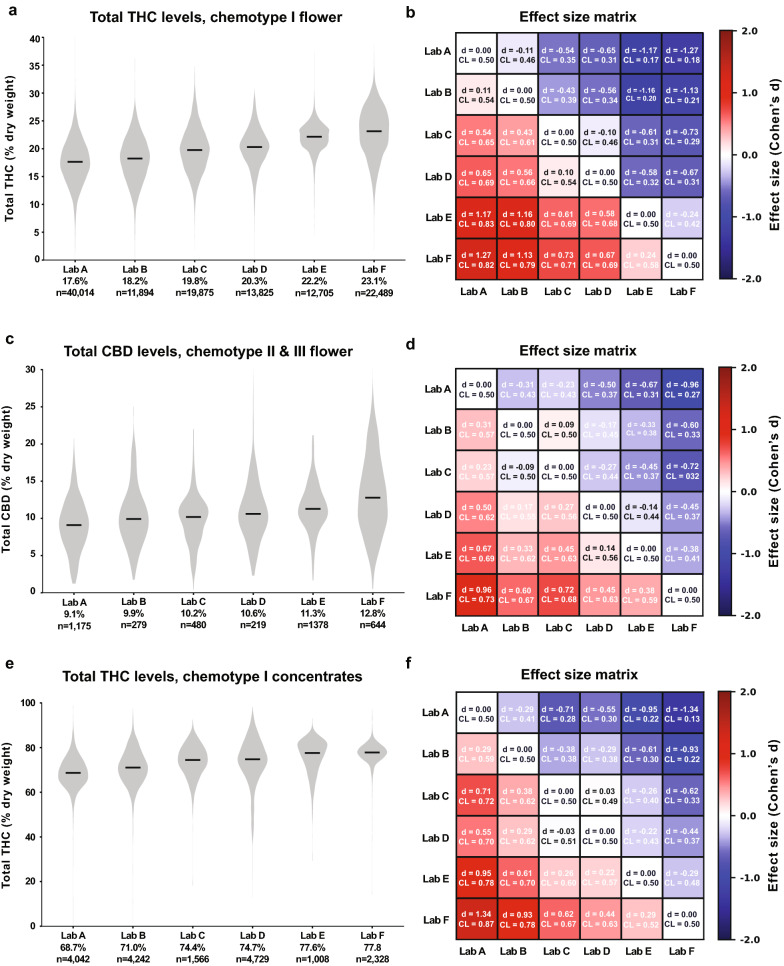
Total THC and CBD Measurements Differ Between Labs Across Chemotypes and Product Categories. Left column: Violin plots showing the distribution of total THC or CBD levels across labs A-F. Black lines denote median values, which are printed below the x-axis for each lab. Right column: Effect size matrices displaying the effect size of pairwise differences in distributions between labs. Matrices are color-coded according to one measure of effect size (Cohen’s d), and a second measure (Common Language) is printed for each comparison.

should read:

“For example, the median total THC content for chemotype I flower products ranged from 17.6% to 23.1% between the labs reporting the lowest and highest THC levels, respectively (Fig. 2a; labs A-F ordered from lowest to highest median reported THC levels).”

“For example, mean THC levels of chemotype I flower for Lab B and Lab A were 18.4% and 17.7%, respectively (Fig. 2a and b). While this difference was highly significant due to the large sample sizes, the effect size was small (d = 0.13; see Methods).”

should read:

“For example, mean THC levels of chemotype I flower for Lab B and Lab A were 18.1% and 17.6%, respectively (Fig. 2a and b). While this difference was highly significant due to the large sample sizes, the effect size was small (d = 0.11; see Methods).”

“In contrast, when comparing Lab F to Lab A, which reported the highest mean THC levels, the effect size was considerably larger (d = 1.28, CL = 0.82).”

should read:

“In contrast, when comparing Lab F to Lab A, which reported the highest mean THC levels, the effect size was considerably larger (d = 1.27, CL = 0.82).”

In the “Interlab Differences Persist After Controlling for Plausible Confounds” section,

“Four separate regression models were estimated: (1) THC levels in chemotype I flower products (n = 161,933); (2) THC levels in chemotype I concentrate products (n = 33,888); (3) CBD levels in chemotype II and III flower products (n = 4,661); and (4) CBD levels in chemotype II and III concentrate products (n = 2,156).”

should read:

“Four separate regression models were estimated: (1) THC levels in chemotype I flower products (n = 115,626); (2) THC levels in chemotype I concentrate products (n = 24,065); (3) CBD levels in chemotype II and III flower products (n = 2,955); and (4) CBD levels in chemotype II and III concentrate products (n = 1,125).”

In the “Changes in THC Content of Commercial Cannabis Products Over Time” section,

“Again, large sample sizes allowed small differences in mean THC levels to reach statistical significance for all pairwise comparisons (p < 0.001, Mann–Whitney U test), except 2015 to 2016 for the HTR cohort (p = 0.334, Fig. 5d).”

should read:

“Again, large sample sizes allowed small differences in mean THC levels to reach statistical significance for all pairwise comparisons (p < 0.001, Mann–Whitney U test).”

In the “THC Content Across Popular Commercial Categories: Indica, Sativa, and Hybrid” section,

“This matching process yielded 166,594 flower results for analysis: 42,711 indica (25.6%), 31,822 sativa (19.1%), and 92,061 hybrid (55.3%) products.”

should read:

“This matching process yielded 118,581 flower results for analysis: 30,754 indica (25.9%), 22,676 sativa (19.1%), and 65,151 hybrid (54.9%) products.”

“The model indicates that hybrid strains have modestly greater THC content, on average, than either indica or sativa strains (Fig. 6e; hybrid vs indica: 1.22%, p < 0.001; hybrid vs sativa: 0.89%, p < 0.01). The difference in THC between sativa and indica could not be distinguished from sampling variability (sativa vs indica: 0.33%, p = 0.230). Moreover, the indica, sativa, hybrid distinction explained only a tiny fraction of THC variability between flower samples (r^2^ = 0.016), and the differences in mean THC content had modest effect sizes (Hybrid vs Sativa: Cohen’s d = 0.283; Hybrid vs Indica: Cohen’s d = 0.206; Indica vs Sativa: Cohen’s d =  − 0.079). An analogous test for variability in CBD content across strain categories among chemotype 2 and chemotype 3 flower yielded similar results (Fig. 6f; hybrid vs indica: 2.17%, p < 0.01; hybrid vs sativa: 0.90%, p = 0.261; sativa vs indica: 1.26%, p < 0.05) with modest effect sizes (Hybrid vs Indica: Cohen’s *d* = 0.148, Hybrid vs Sativa: Cohen’s *d* = 0.068, Sativa vs Indica: Cohen’s *d* = 0.268).”

should read:

“The model indicates that hybrid strains have modestly greater THC content, on average, than either indica or sativa strains (Fig. 6e; hybrid vs indica: 1.19%, p < 0.001; hybrid vs sativa: 0.75%, p < 0.05). The difference in THC between sativa and indica could not be distinguished from sampling variability (sativa vs indica: 0.45%, p = 0.116). Moreover, the indica, sativa, hybrid distinction explained only a tiny fraction of THC variability between flower samples ( r^2^ = 0.014), and the differences in mean THC content had modest effect sizes (Hybrid vs Sativa: Cohen’s d = 0.273; Hybrid vs Indica: Cohen’s d = 0.172; Indica vs Sativa: Cohen’s d =  − 0.104). An analogous test for variability in CBD content across strain categories among chemotype 2 and chemotype 3 flower yielded similar results (hybrid vs indica: 2.10%, p < 0.01; hybrid vs sativa: 0.86%, p = 0.247; sativa vs indica: 1.24%, p < 0.05) with modest effect sizes (Hybrid vs Indica: Cohen’s d = 0.102, Hybrid vs Sativa: Cohen’s d = 0.022, Sativa vs Indica: Cohen’s d = 0.083).”

“However, performing the same regression solely using flower products from Lab F (n = 22,049), which reports the highest mean THC levels, would not detect the higher average THC levels of hybrid flowers (hybrid vs indica: 0.47%, p = 0.21; hybrid vs sativa: 0.28%, p = 0.425). Repeating the analysis with solely flower products from Lab A (n = 50,610), the lab reporting the lowest mean THC levels, replicates the overall result, with hybrids having slightly higher THC than indica (1.13%, p < 0.001) and sativa (0.80%, p < 0.01).”

should read:

“However, performing the same regression solely using flower products from Lab F (n = 18,634), which reports the highest mean THC levels, would not detect the higher average THC levels of hybrid flowers (hybrid vs indica: 0.57%, p = 0.123; hybrid vs sativa: 0.25%, p = 0.492). Repeating the analysis with solely flower products from Lab A (n = 32,274), the lab reporting the lowest mean THC levels, replicates the overall result, with hybrids having slightly higher THC than indica (1.07%, p < 0.001) and sativa (0.75%, p < 0.01).”

In the “Cannabinoid Variation Within and Across Popular Commercial Strain Names” section,

“Across all labs, the pre-filtered strain ICC was 0.57 (omitting results with zero reported CBD or THC) or 0.41 (coercing results with zero reported CBD to 3.5 and zero THC to − 2.0). After filtering, the ICCs increase, respectively, to about 0.71 and 0.51. This overall test conceals significant variation between labs.”

should read:

“Across all labs, the pre-filtered strain ICC was 0.54 (omitting results with zero reported CBD or THC) or 0.37 (coercing results with zero reported CBD to 3.5 and zero THC to − 2.0). After filtering, the ICCs increase, respectively, to about 0.68 and 0.47. This overall test conceals significant variation between labs.”

“For Lab F, the lab with the highest proportion of zero CBD results, the ICC was very sensitive to the handling of missing data; the ICC was 0.76 and 0.48, respectively, before filtering, and 0.92 and 0.53, after filtering. In contrast, Lab A, which had few zero CBD values, had a stable ICC across both methods, with pre-filtered ICCs of 0.65 and 0.64, respectively, and post-filtering ICCs of 0.79 and 0.78.”

should read:

“For Lab F, the lab with the highest proportion of zero CBD results, the ICC was very sensitive to the handling of missing data; the ICC was 0.74 and 0.50, respectively, before filtering, and 0.90 and 0.59, after filtering. In contrast, Lab A, which had few zero CBD values, had a stable ICC across both methods, with pre-filtered ICCs of 0.62 and 0.60, respectively, and post-filtering ICCs of 0.76 and 0.74.”

In the Discussion section,

“In our analyses of Washington labs, median THC levels for chemotype I flower varied considerably (Fig. 2), ranging from 17.7% to 23.2%.”

should read:

“In our analyses of Washington labs, median THC levels for chemotype I flower varied considerably (Fig. 2), ranging from 17.6% to 23.1%.”

“Restricted to the lab reporting the lowest THC levels for chemotype I flower (median = 17.7%), the 99th percentile THC level is 27.0%, compared to 31.8% for the lab reporting the highest THC levels.”

should read:

“Restricted to the lab reporting the lowest THC levels for chemotype I flower (median = 17.6%), the 99th percentile THC level is 26.9%, compared to 31.7% for the lab reporting the highest THC levels.”

“In general, our results do not suggest that flower samples labeled as indica, sativa, and hybrid differ substantially in terms of total THC content or THC:CBD profiles, as the indica/sativa/hybrid typology accounted for only about 1% of the relative variability in THC content (r^2^ = 0.016).”

should read:

“In general, our results do not suggest that flower samples labeled as indica, sativa, and hybrid differ substantially in terms of total THC content or THC:CBD profiles, as the indica/sativa/hybrid typology accounted for only about 1% of the relative variability in THC content (r^2^ = 0.014).”

In the “Data” section of the Methods, under “Washington I-502 Cannabis Test Data”,

“304,123 test results”.

should read:

“215,285 test results”.

In the “Analytic Methods” section of the Methods, under “Matching Strain Names”,

“Through this process, used to minimize the number of false positive matches, 214,747 (70.6%) results were matched to Leafly strains, including 166,594 for flower samples and the remainder for other product types.”

should read:

“Through this process, used to minimize the number of false positive matches, 153,363 (71.2%) results were matched to Leafly strains, including 118,581 for flower samples and the remainder for other product types.”

In the legend for Figure 1,

“Data are displayed for measurements batched across all Labs A-F (panels a-b; n = 175,136), for the lab reporting the lowest mean total THC levels (Lab A; panels c-d; n = 62,719), and the lab reporting the highest mean total THC levels (Lab F; panels e–f; n = 26,664).”

should read:

“Data are displayed for measurements batched across all Labs A-F (panels a-b; n = 123,977), for the lab reporting the lowest mean total THC levels (Lab A; panels c-d; n = 41,189), and the lab reporting the highest mean total THC levels (Lab F; panels e–f; n = 23,133).”

In the legend for Figure 3,

**Figure 3:**
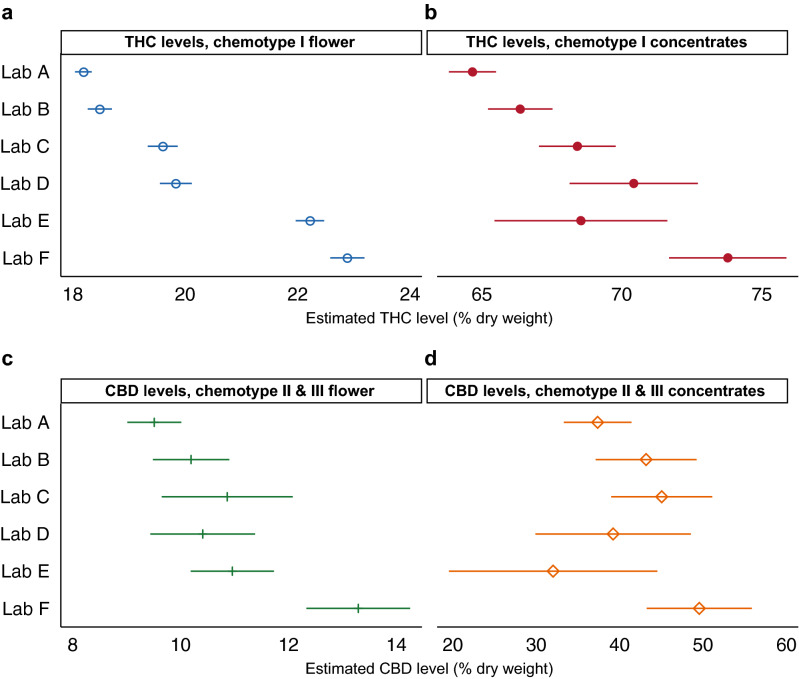
THC and CBD Levels Vary Between Labs After Controlling for Plausible Confounds. Average predicted values (+/− 99% confidence intervals) are shown, by lab, for (**a**) THC levels in chemotype I flower products (n = 115,626); (**b**) THC levels in chemotype I concentrate products (n = 24,065); (**c**) CBD levels in chemotype II and III flower products (n = 2,955); and (**d**) CBD levels in chemotype II and III concentrate products (n = 1,125) after adjusting for grower, strain-name, and time of measurement. Predicted values were generated from fixed-effects regressions with cluster-robust standard errors (see Methods).

“Average predicted values (+/− 99% confidence intervals) are shown, by lab, for (**a**) THC levels in chemotype I flower products (n = 161,933); (**b**) THC levels in chemotype I concentrate products (n = 33,888); (**c**) CBD levels in chemotype II and III flower products (n = 4,661); and (**d**) CBD levels in chemotype II and III concentrate products (n = 2,156) after adjusting for grower, strain-name, and time of measurement.”

should read:

“Average predicted values (+/− 99% confidence intervals) are shown, by lab, for (**a**) THC levels in chemotype I flower products (n = 115,626); (**b**) THC levels in chemotype I concentrate products (n = 24,065); (**c**) CBD levels in chemotype II and III flower products (n = 2,955); and (**d**) CBD levels in chemotype II and III concentrate products (n = 1,125) after adjusting for grower, strain-name, and time of measurement.”

The correct Figures 1, 2, 3, 4, 5, 6, 7 and 8 appear below.

**Figure 4:**
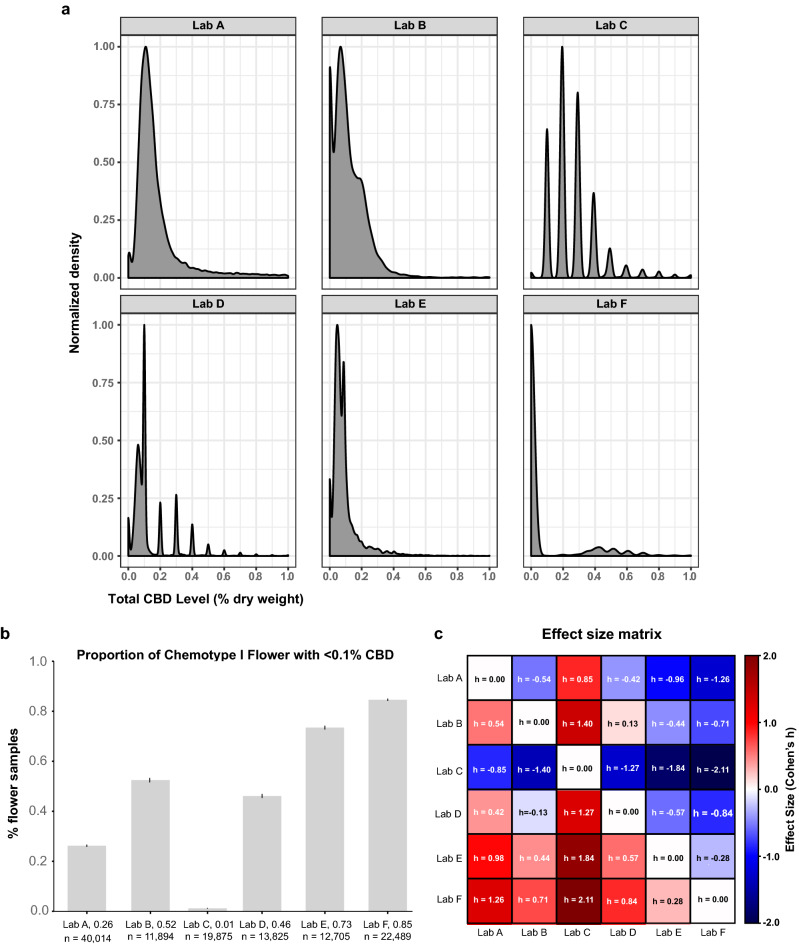
Labs differ in the propensity to detect low levels of CBD in chemotype I flower. (**a**) Kernel density plots of each lab’s distribution of total CBD levels below 1.0% dry weight for chemotype I flower (y-axis scaled to one). Most labs show a local maximum near 0.1% total CBD, which is a commonly reported LOQ. (**b**) Fraction of chemotype I flower with total CBD levels below 0.1% dry weight. Bars indicate proportions +/− 95% CI for a binomial proportion. (**c**) Effect size matrix indicating the magnitude of interlab differences shown in panel B. Effect size is quantified as Cohen’s h (see Methods).

**Figure 5:**
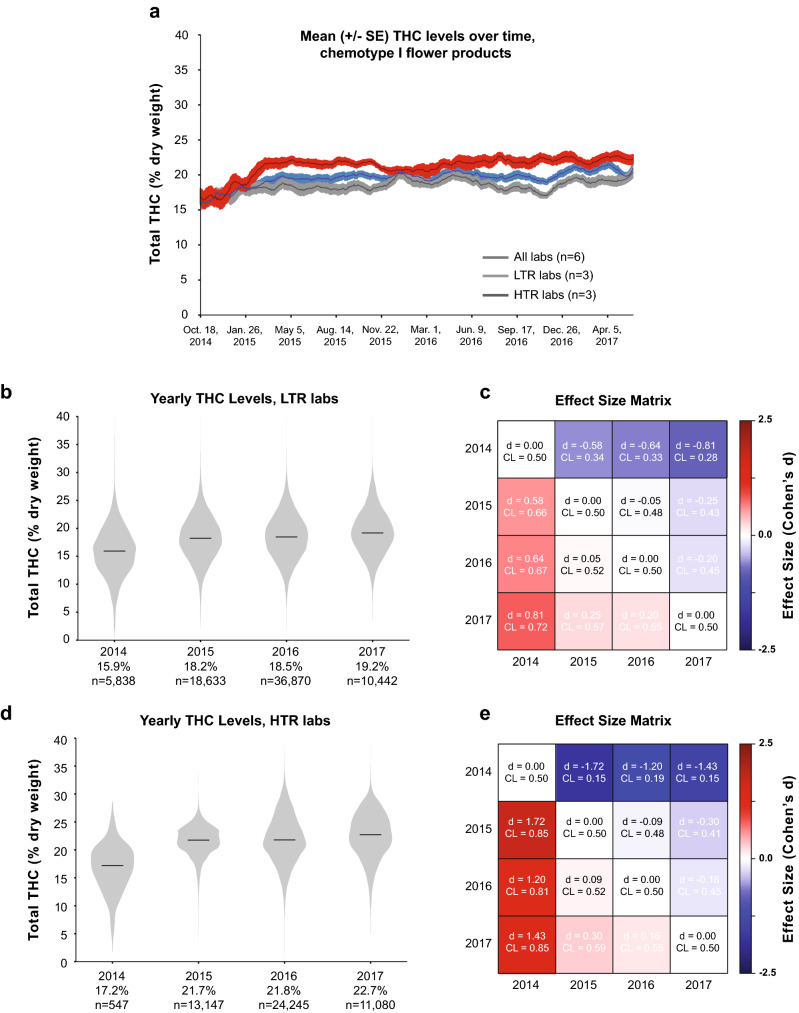
Mean THC Levels for Chemotype I Flower Products Over Time. (**a**) Total THC levels over time averaged across all labs or those reporting the highest or lowest mean THC levels. (**b**) Distribution of THC levels for each year on record for low THC reporting (LTR) labs. (**c**) Effect size matrix quantifying the mean difference in THC levels across years for LTR labs. (**d**) Distribution of THC levels for each year for high THC reporting (HTR) labs, and (**e**) the effect size matrix quantifying the magnitude of yearly differences.

**Figure 6:**
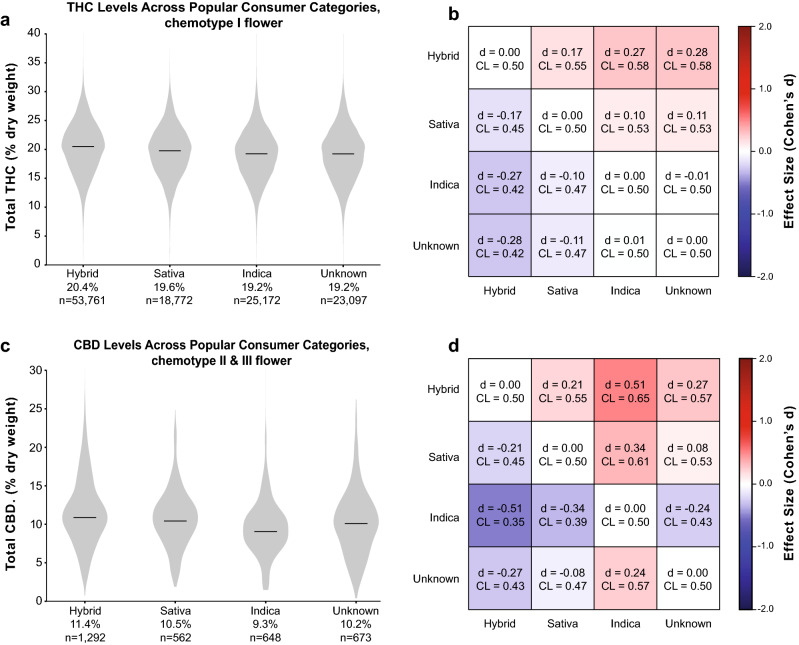
Total THC and CBD Levels Across Popular Consumer Strain Categories for Flower Products. (**a**) Distribution of THC levels across popular strain categories for chemotype I flower and (**b**) effect size matrix quantifying the magnitude of differences between them. (**c**) Distribution of CBD levels across the same categories for chemotype II and III flower and (**d**) effect size matrix quantifying the magnitude of differences between them.

**Figure 7:**
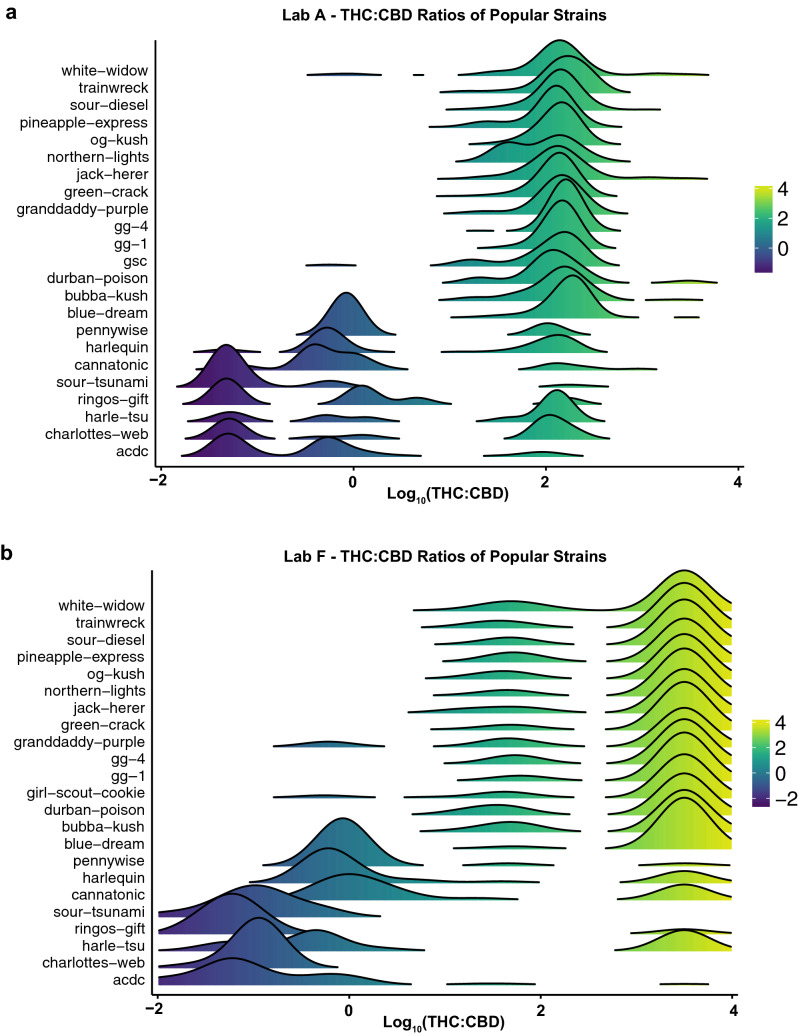
Distribution of THC-to-CBD Ratios Vary Across Popular Commercial Strain Names and Between Labs. THC-to-CBD ratios plotted on a logarithmic scale for cannabis flower samples across twenty-three popular commercial strain names for the single lab (Lab A) reporting the lowest (**a**) and the single lab (Lab F) reporting the highest (**b**) overall THC levels for cannabis flower.

**Figure 8:**
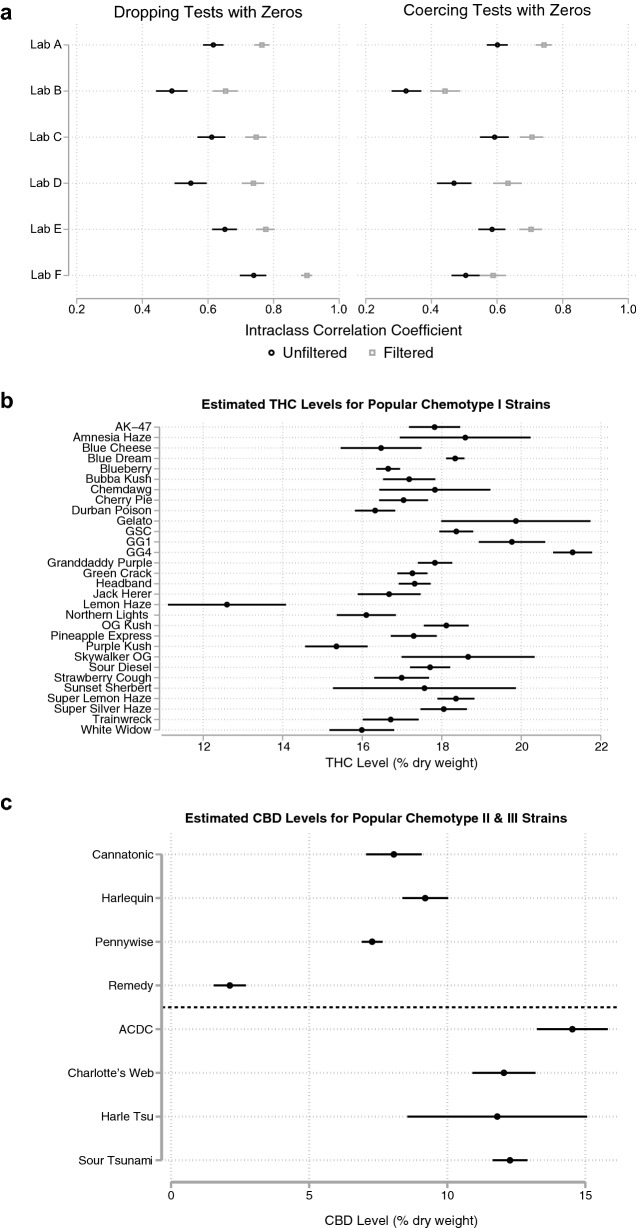
Popular Strain Names as Signal for THC and CBD Content. (**a**) Proportion of variation in log_10_ THC:CBD ratio explained by popular strain names (Intraclass Correlation Coefficient). 99% CIs are shown, by lab, before (black circles) and after (gray squares) filtering test results by the modal chemotype of each strain name. The ICC is shown both for dropping values for which 0% CBD or 0% THC is reported (left) and coercing cannabinoid ratios for these tests (see Methods). (**b**) Mean THC level of popular chemotype I strains. 99% CIs are shown after filtering by modal chemotype, for the lab reporting the lowest THC levels. (**c**) Mean CBD levels for popular chemotype II (above dotted line) and chemotype III (below dotted line) strain names. Results shown for the lab reporting the lowest mean THC levels.

The Supplementary Dataset 1 linked to this correction has been updated from the version published with the original paper, with duplicated rows removed.

The Supplemental Figures linked to this correction have been updated from the versions published with the original paper.

The duplicated entries were brought to our attention by Dr. Jim MacRae, a cannabis industry analyst and consultant who downloaded our open access dataset. We thank him for reviewing the dataset and bringing it to our attention, and for his work in the public sphere leveraging the I-502 dataset to shed light on potency and safety issues in cannabis laboratory testing in the State of Washington. Dr. MacRae shares many of his insights in the blog section of his website: https://www.straightlineanalytics.biz/hi-blog

## Supplementary information

Supplementary Information.

Supplementary Dataset.

