# Supplementary material for: The Cannabinoid Content of Legal Cannabis in Washington State Varies Systematically Across Testing Facilities and Popular Consumer Products
Source: Sci Rep. 2018 Mar 14;8:4519. doi: 10.1038/s41598-018-22755-2 (PMC5852027; doi:10.1038/s41598-018-22755-2)
Supplement: Supplementary file 1 — Supplemental Figures [file 41598_2018_22755_MOESM1_ESM.pdf]

The Cannabinoid Content of Legal Cannabis in Washington State Varies Systematically Across Testing Facilities and Popular Consumer Products

Nick Jikomes[1*] & Michael Zoorob[2*]

[1]Leafly Holdings, Inc., Division of Data Science, Seattle, WA 98104, USA. [2]Harvard University, Department of Government, Cambridge, MA 02138, USA. *These authors contributed equally to this work. Correspondence and requests for materials should be addressed to N.J. (email: njikomes@gmail.com)

Supplemental Figure Legends

Figure S1: THC:CBD Distributions of Flower Products Vary Widely Across Labs. Histograms showing the distribution of THC:CBD for flower products measured by six I-502 certified laboratories between 2014-2017. (a) Lab A (n=62,719); (b) Lab B (n=17,786); (c) Lab C (n=31,945); (d) Lab D (n=22,234); (e) Lab E (n=13,788); (f) Lab F (n=26,664).

Figure S2: THC:CBD Distributions of Concentrate Products Vary Widely Across Labs. Histograms showing the distribution of THC:CBD for concentrates measured by the six Washington state laboratories between 2014-2017. (a) Lab A (n=6,871); (b) Lab B (n=7,128); (c) Lab C (n=2,793); (d) Lab D (n=8,727); (e) Lab E (n=1,126); (f) Lab F (n=3,152).

Figure S3: Mean THC Levels for Chemotype I Concentrates Over Time. (a) Total THC levels over time averaged across all labs (n=6) vs low THC reporting (LTR) and high THC reporting (HTR) labs (n=3 each). (b) Distribution of THC levels for each year on record for the three labs reporting the lowest mean THC levels. (c) Effect size matrix quantifying the mean difference in THC levels across years for low-THC labs. (d) Distribution of THC levels for each year for the three labs reporting the highest mean THC levels, and (e) the effect size matrix quantifying the magnitude of yearly differences.

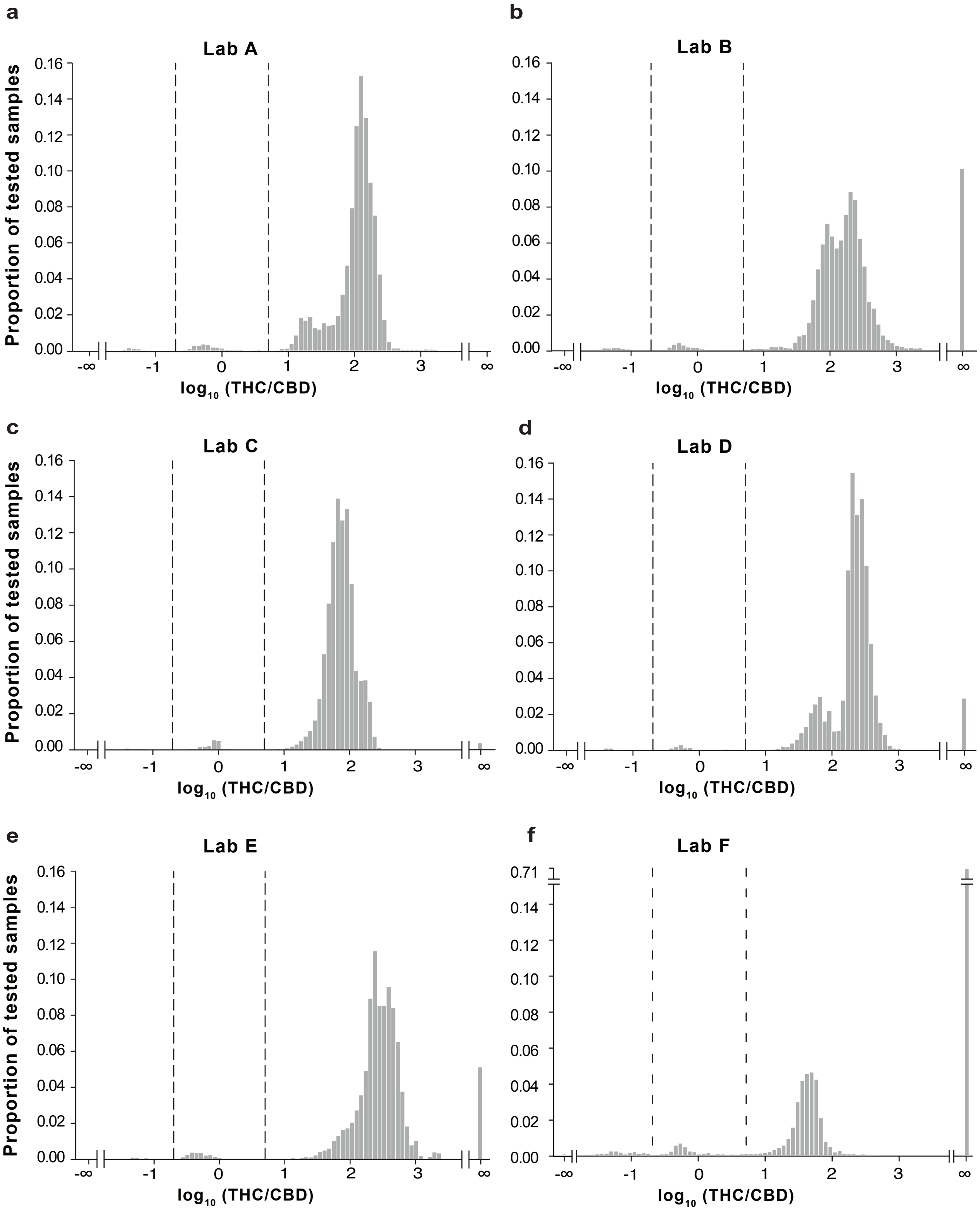

a
Lab A
b
Lab B
c
Lab C
d
Lab D
e
Lab E
f
Lab F
Proportion of tested samples
0.16
0.14
0.12
0.10
0.08
0.06
0.04
0.02
0.00
0.71
-∞
-1
0
1
2
3
∞
log10 (THC/CBD)

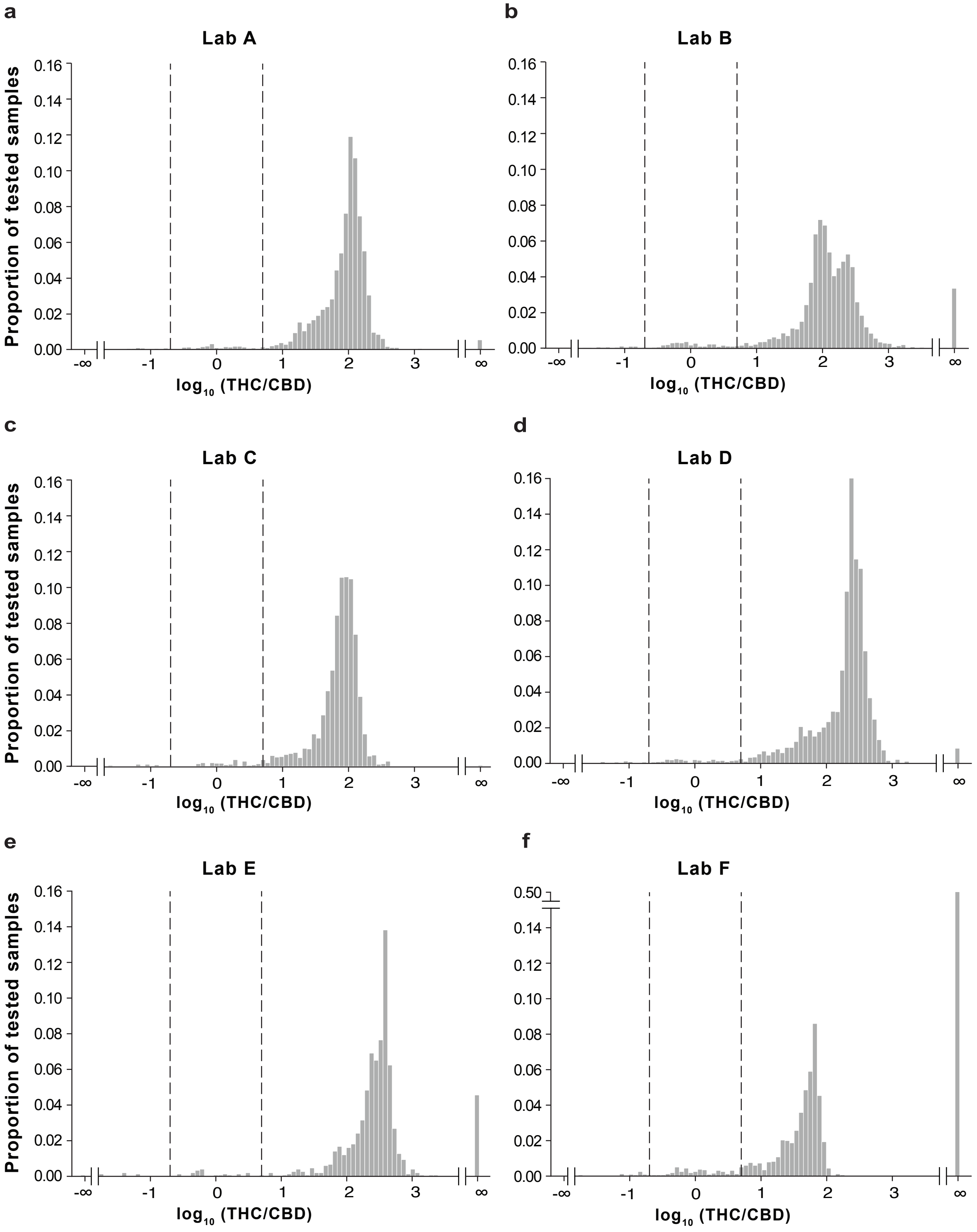
a
Lab A
b
Lab B
c
Lab C
d
Lab D
e
Lab E
f
Lab F
Proportion of tested samples
0.16
0.14
0.12
0.10
0.08
0.06
0.04
0.02
0.00
0.50
-∞
-1
0
1
2
3
∞
log10 (THC/CBD)

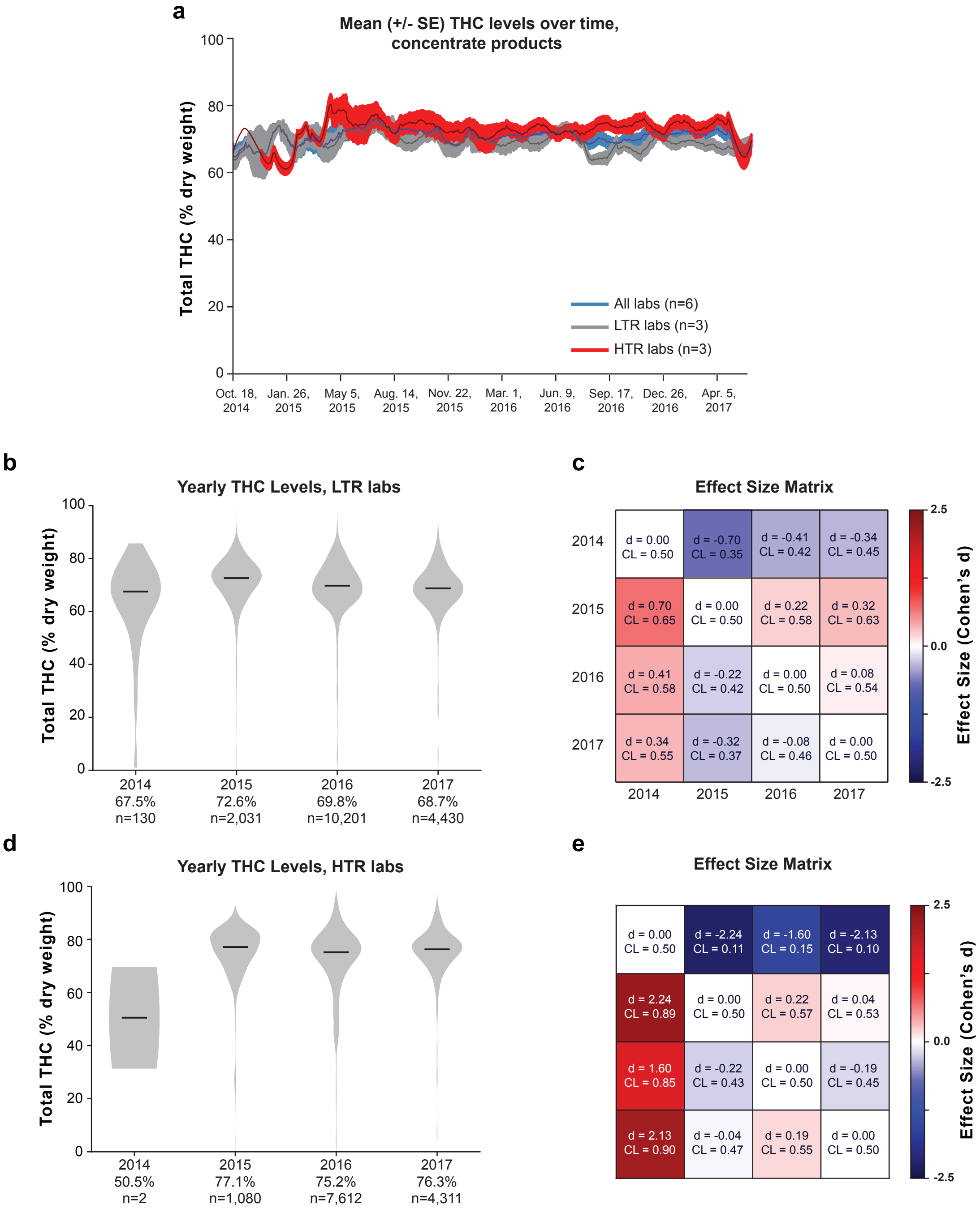

a
Mean (+/- SE) THC levels over time, concentrate products
Total THC (% dry weight)
100
80
60
40
20
0
All labs (n=6)
LTR labs (n=3)
HTR labs (n=3)
Oct. 18, 2014
Jan. 26, 2015
May 5, 2015
Aug. 14, 2015
Nov. 22, 2015
Mar. 1, 2016
Jun. 9, 2016
Sep. 17, 2016
Dec. 26, 2016
Apr. 5, 2017
b
Yearly THC Levels, LTR labs
Total THC (% dry weight)
2014 67.5% n=130
2015 72.6% n=2,031
2016 69.8% n=10,201
2017 68.7% n=4,430
c
Effect Size Matrix
2014: d = 0.00 CL = 0.50 | d = -0.70 CL = 0.35 | d = -0.41 CL = 0.42 | d = -0.34 CL = 0.45
2015: d = 0.70 CL = 0.65 | d = 0.00 CL = 0.50 | d = 0.22 CL = 0.58 | d = 0.32 CL = 0.63
2016: d = 0.41 CL = 0.58 | d = -0.22 CL = 0.42 | d = 0.00 CL = 0.50 | d = 0.08 CL = 0.54
2017: d = 0.34 CL = 0.55 | d = -0.32 CL = 0.37 | d = -0.08 CL = 0.46 | d = 0.00 CL = 0.50
2014 2015 2016 2017
Effect Size (Cohen's d)
2.5
0.0
-2.5
d
Yearly THC Levels, HTR labs
Total THC (% dry weight)
2014 50.5% n=2
2015 77.1% n=1,080
2016 75.2% n=7,612
2017 76.3% n=4,311
e
Effect Size Matrix
d = 0.00 CL = 0.50 | d = -2.24 CL = 0.11 | d = -1.60 CL = 0.15 | d = -2.13 CL = 0.10
d = 2.24 CL = 0.89 | d = 0.00 CL = 0.50 | d = 0.22 CL = 0.57 | d = 0.04 CL = 0.53
d = 1.60 CL = 0.85 | d = -0.22 CL = 0.43 | d = 0.00 CL = 0.50 | d = -0.19 CL = 0.45
d = 2.13 CL = 0.90 | d = -0.04 CL = 0.47 | d = 0.19 CL = 0.55 | d = 0.00 CL = 0.50
Effect Size (Cohen's d)
2.5
0.0
-2.5
