# Supplementary figures and images for: Author Correction: The Cannabinoid Content of Legal Cannabis in Washington State Varies Systematically Across Testing Facilities and Popular Consumer Products
Source: Sci Rep. 2020 Aug 27;10:14406. doi: 10.1038/s41598-020-69680-x (PMC7506002; doi:10.1038/s41598-020-69680-x)

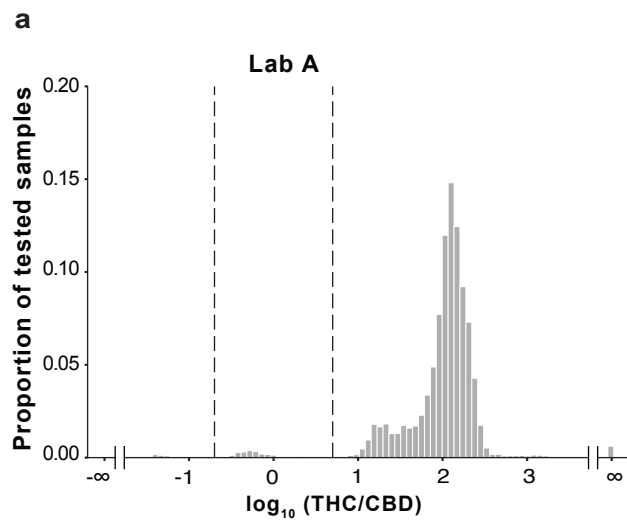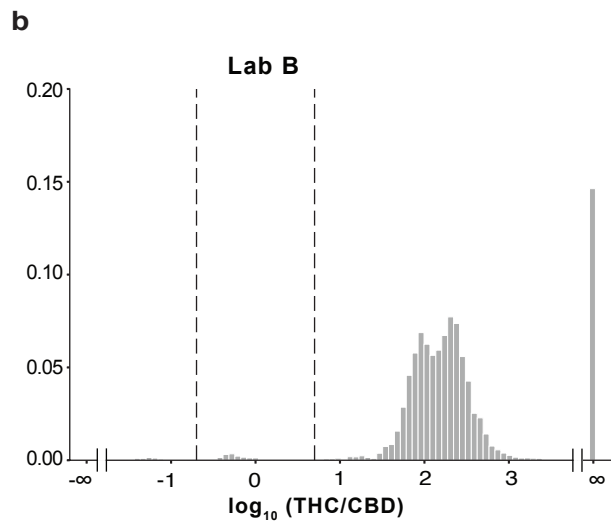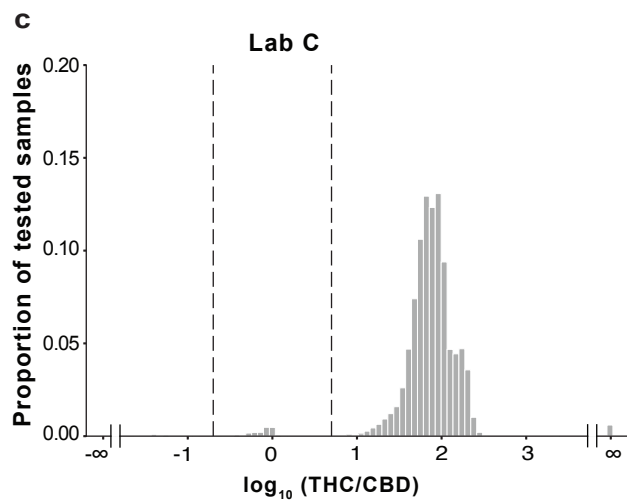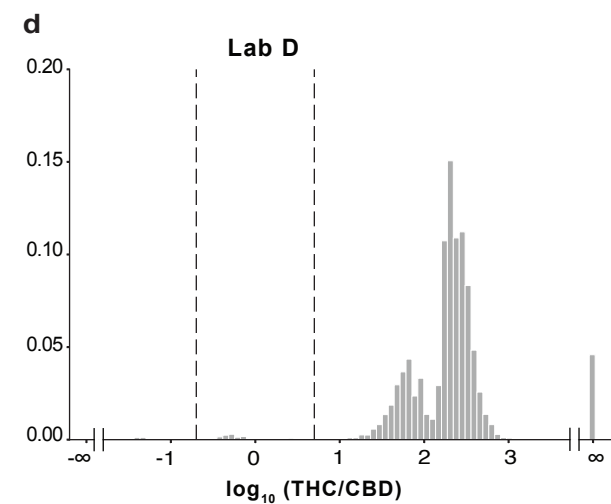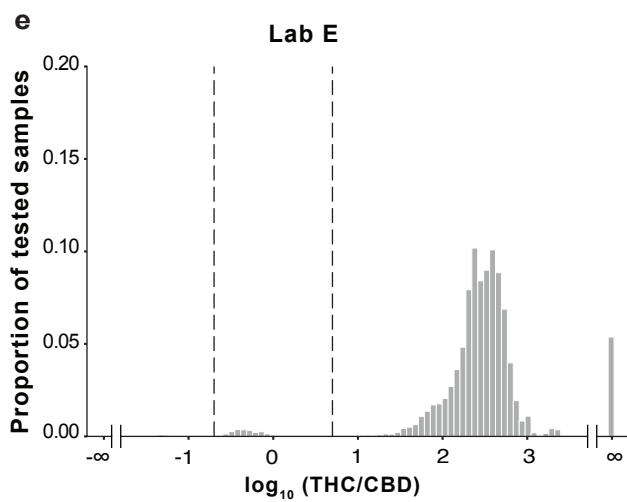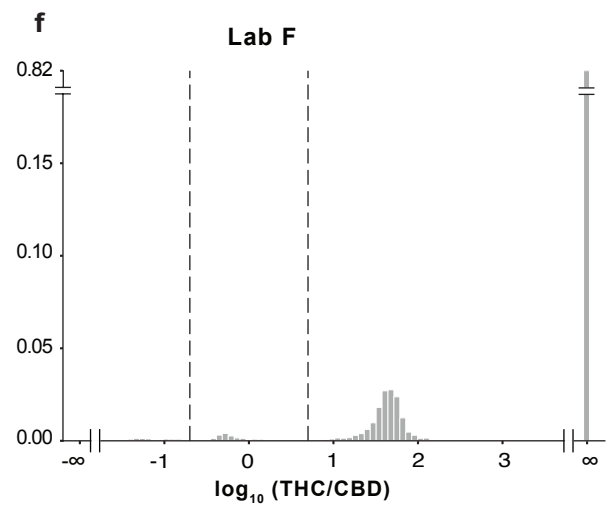

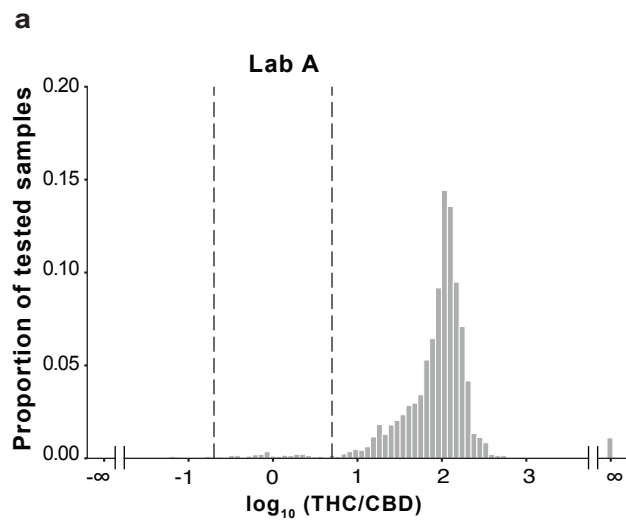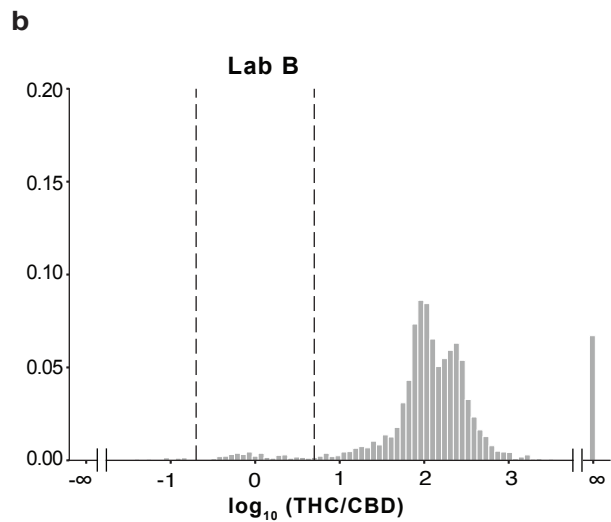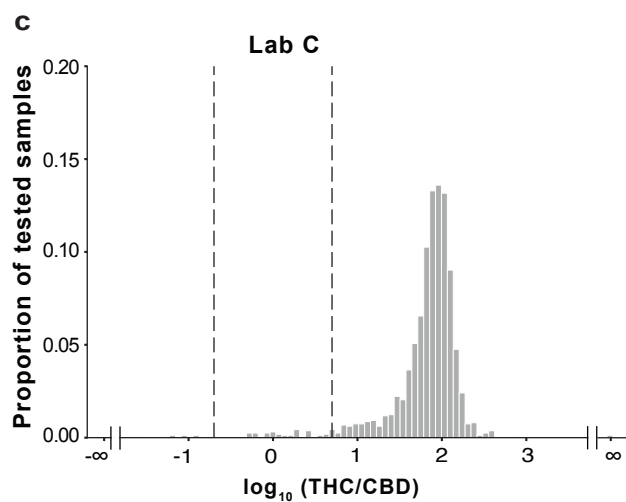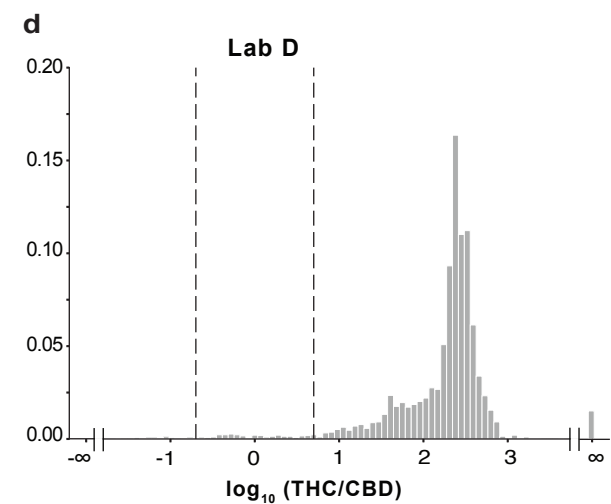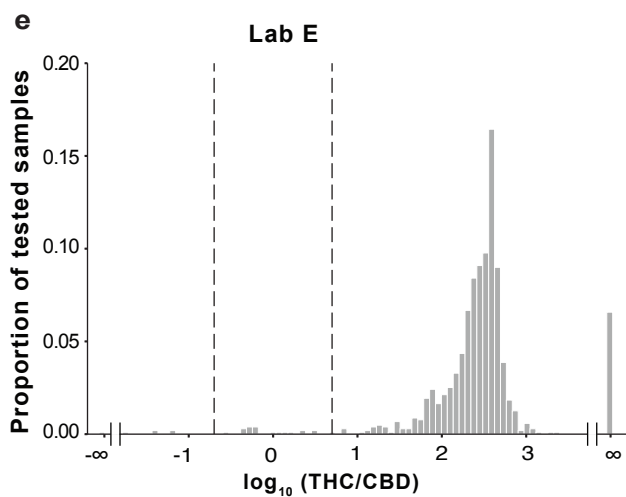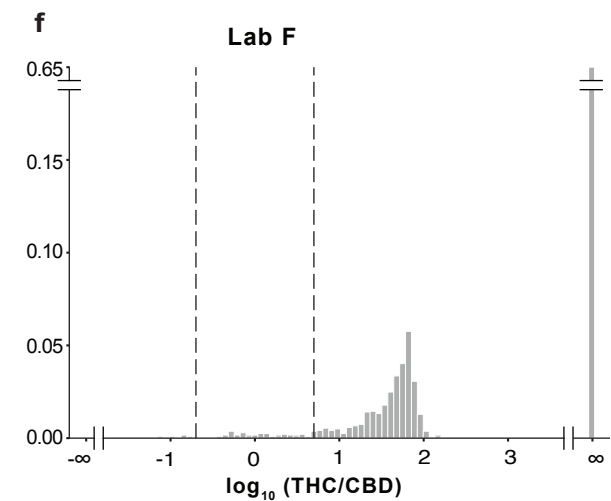

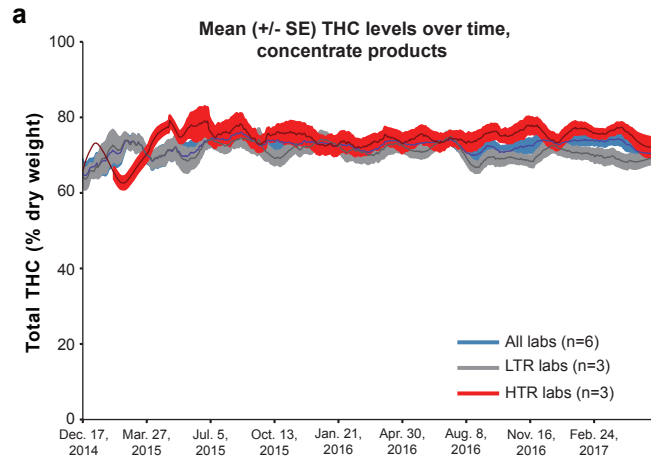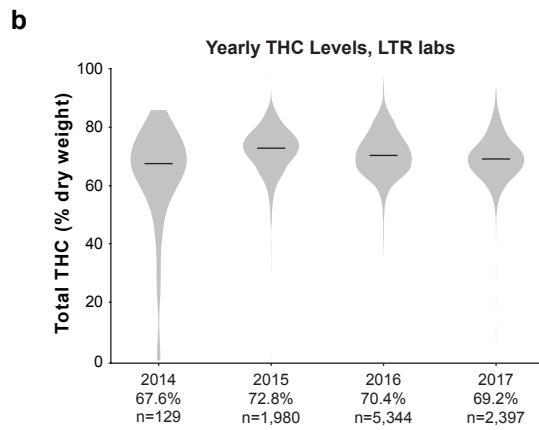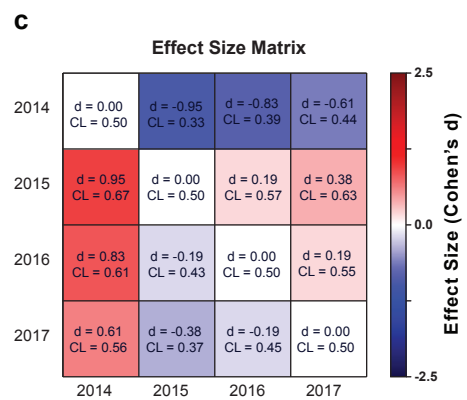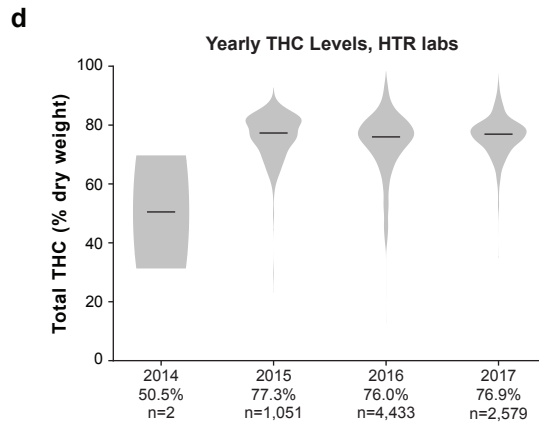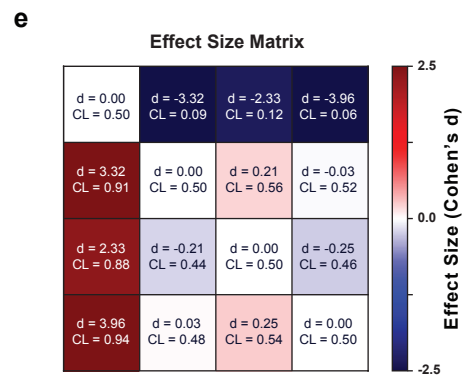

Supplement: Supplementary file 1 — Supplementary Information. [file 41598_2020_69680_MOESM1_ESM.pdf]
